# Physics or biology? Persistent chlorophyll accumulation in a shallow coastal sea explained by pathogens and carnivorous grazing

**DOI:** 10.1371/journal.pone.0212143

**Published:** 2019-02-22

**Authors:** Kai W. Wirtz

**Affiliations:** Institute of Coastal Research, Helmholtz-Zentrum Geesthacht, Geesthacht, Germany; Universita del Salento, ITALY

## Abstract

One of the most striking patterns at the land–ocean interface is the massive increase of chlorophyll-a (CHL) from continental shelves towards the coast, a phenomenon that is classically linked to physical features. Here I propose that the coastal–offshore CHL gradient in a shallow sea has biological origins related to phytoplankton mortality that are neglected in state-of-the-art biogeochemical models. I integrate a trait-based ecosystem model into a modular coupling framework that is applied to the southern North Sea (SNS). The coupled model very well reproduces daily, seasonal and inter-annual (2000-2014) dynamics and meso-scale patterns in macronutrients, zooplankton biomass, and CHL as observed *in situ* and by remote sensors. Numerical experiments reveal that coast–offshore CHL gradients may predominantly arise from a trophic effect as resolved by an increase in carnivorous grazing towards shallow waters. This carnivory gradient reflects higher near-coast abundance of juvenile fish and benthic filter feeders. Furthermore, the temporal evolution of CHL can be much affected by viral infection as a fast-responding loss process at intermediate to high phytoplankton concentrations. Viral control in the model also prevents excessive and unrealistic blooms during late spring. Herbivores as often only ecological factor considered for explaining the spatio-temporal phytoplankton distribution are in this study supplemented by pathogens as well as pelagic and benthic carnivores as powerful agents, which are barely represented in current modeling but can mediate physical drivers of coastal ecosystems.

## Introduction

Marine primary production by unicellular autotrophs makes the fundamental basis of both biogeochemical cycling and oceanic to coastal food-webs [1, 2, 3]. Hindcasting the observed spatio-temporal distribution of primary producers thus defines a critical test of ecosystem models as a pre-condition for ongoing model–based estimates of how marine ecosystems respond to climate change or direct anthropogenic stressors. Model hindcasts also reflect the state of our mechanistic understanding of phytoplankton ecophysiology and pelagic ecosystem dynamics and, in total, pose a quintessential challenge in marine research. The distribution of phytoplankton in time and space has recently gained greater visibility through marine–coastal observatories and monitoring networks (e.g., www.jerico-ri.eu, or [4]) and remotely detected changes in chlorophyll-a (CHL) concentration, a proxy for phytoplankton abundance or, better, potential photosynthetic activity [2, 5, 6, 3, 7]. This rise in the number and quality of global and regional data-sets and concomitant disclosure of multiscale variability in CHL has puzzled our quantitative understanding rather than making model calculations more reliable, mostly because of the ubiquitous divergence and low correlation between these patterns and the results of ecosystem models [8, 9, 10, 11]. This is especially evident for shelf and coastal seas where primary production is subject to many additional drivers that are negligible in the global ocean such as tidal currents or benthic and terrestrial nutrient inputs. Previous modeling studies in general succeeded in reproducing climatological trends but often showed a decreasing skill from physical, chemical towards biological variables and also from offshore regions to the coastal zone [12].

### Coastal gradient in chlorophyll-a

This study focusses on the most ubiquitous, persistent, and striking pattern that to date challenges current modeling: Global and regional analyses of satellite data point to a massive increase of CHL from continental shelves towards the coast [13, 14, 5, 7]. In steep bathymetries or at the shelf edge, coastal upwelling replenishes nutrients potentially stimulating phytoplankton growth. Shallow bathymetries can feature riverine nutrient sources and strong benthic-pelagic coupling [15] and/or remineralization of degrading particles trapped by residual cross-shore circulation [16]. However, in shallow waters effective light availability much decreases due to high turbidity while inorganic nutrients are depleted during summer [17]. Growth rates should therefore decline towards the coast at least in the summer period such that the observed CHL accumulation requires explanation.

Here I suggest that the cross-shore CHL gradient at least in relatively shallow waters is ultimately caused by plankton mortality factors which are poorly resolved in state-of-the-art biogeochemical models. Nearly all models spatially and dynamically resolve herbivorous zooplankton as a major mortality factor for phytoplankton, but little attention is often paid to carnivorous grazing. As empirical, system-wide studies on carnivorous grazing rates are rare, most models use a global “closure” parameter and a few resolve a more extended food-chain including fish [18, 19, 20]. However, the first approach ignores potentially large differences in the spatio-temporal distribution of carnivory [21], while the second approach faces the difficulty to represent highly diverse migratory behaviors of fish [22, 23], or other mechanisms that regulate the distribution pattern of carnivores.

A factor even entirely missing in coupled ecosystem models is host-phage dynamics in phytoplankton. Viral infections are very common in unicellular autotrophs, which has already led to the hypothesis that viruses may substantially influence phytoplankton population dynamics and concomitantly also ecosystem dynamics at large [24, 25, 26]. Host-phage interactions in microbes were recently shown to be relevant in biogeochemical cycles simulated by conceptual models [27, 28].

The goal of this study is thus to evaluate the effect of (1) spatial gradients in carnivory and (2) viral dynamics on ecosystem states in a shallow shelf system in general and the CHL accumulation in particular. The study area is the Southern North Sea (SNS), characterized by steep and variable gradients with respect to the major productivity factors nutrients [29, 16] and light climate [30].

To obtain a realistic description of relevant drivers of primary production in the SNS, I make use of the novel framework MOdular System for Shelves and COasts, MOSSCO, [31]. First MOSSCO applications to the SNS revealed validity of the set-setup and individual model components such as for hydrodynamics, biogeochemistry, and sediment dynamics [32, 33, 34]. Here I further extent the Model for Adaptive Ecosystems in Coastal Seas, MAECS, [35, 33], embed it into a MOSSCO set-up, and test the skill of the coupled model using daily CHL maps derived from composite satellite products and long-term time-series for CHL, zooplankton, and inorganic nutrients from seven stations within the SNS. A series of numerical experiments then seeks to unravel how viruses and spatial (non-)uniformity in carnivory contribute to the formation and disappearance of striking CHL structures in a coastal and shelf sea.

## Materials and methods

### Modular data and model system

The framework MOSSCO (www.mossco.de) offers a standardized coupling concept, which facilitates a numerically efficient communication between modules, i.e. model and data components, and allows for their easy exchange [31]. MOSSCO combines and extends the two modeling frameworks ESMF (Earth System Modeling Framework [36]) and FABM (Framework for Aquatic Biogeochemical Models [37]) such that physical, geological, biological, and biogeochemical modules available in one of the two standards can be instantaneously integrated across different Earth System compartments such as the sea floor, water, and atmosphere. MOSSCO and so far realized ensembles of modules indeed put most emphasis on interfaces relevant for coastal and shelf dynamics such as the benthic–pelagic interface in vertical dimension and estuarine–coast laterally [38, 32]. The modularity of MOSSCO specifically facilitated this study because it enabled seamless switches from 0D setups to full 3D setups. Coupled to the FABM-OD driver, the phytoplankton module has been extensively tested and parametrized using a large number of laboratory experiments [35], and similar plausibility tests were performed in 0D during the development of novel biological descriptions, which could then directly been brought to three-dimensional simulations. This study employed MOSSCO v1.0.2, with code available at https://doi.org/10.5281/zenodo.2527238.

### Physical model and set-up

**Fig 1:**
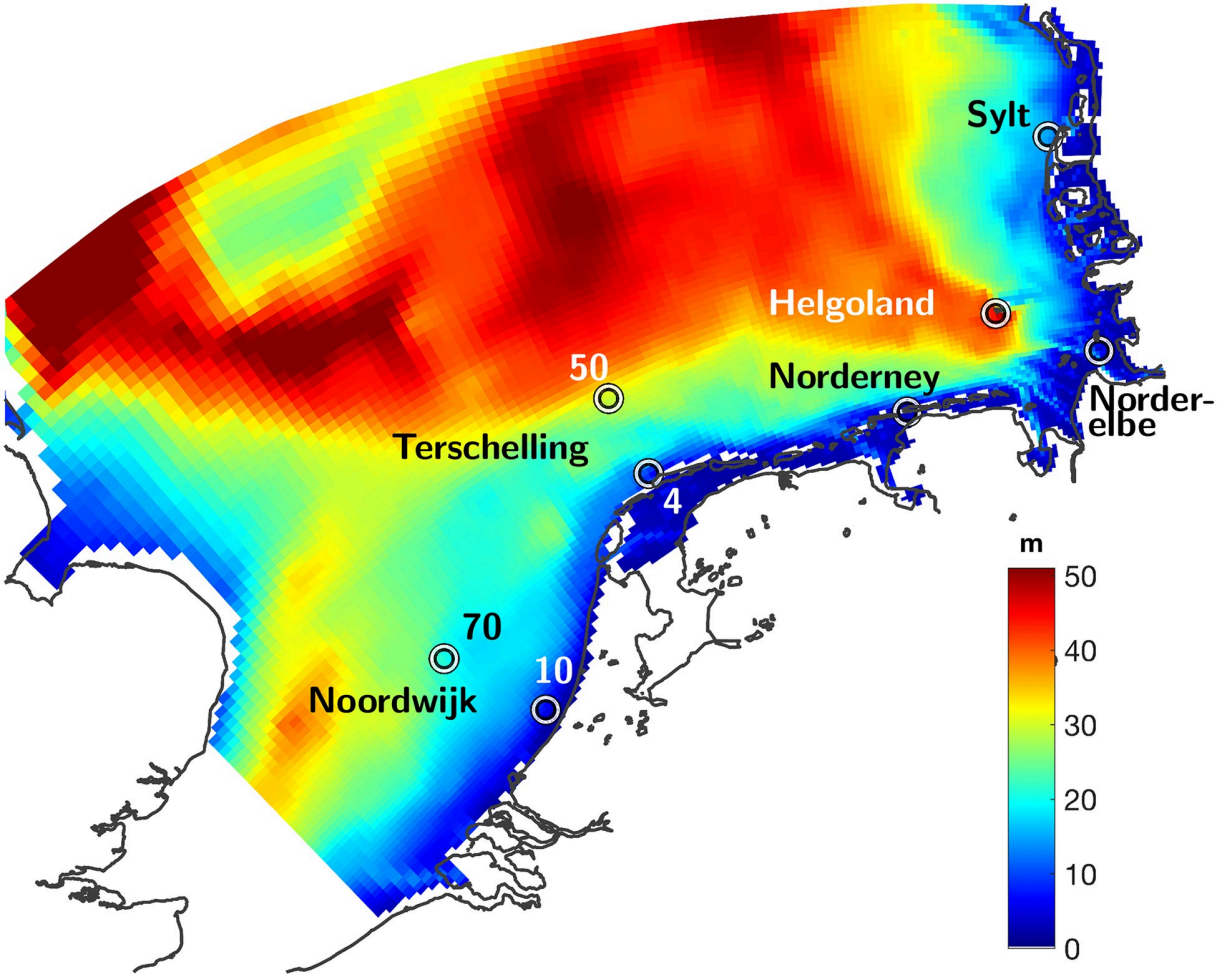
Topography of the model domain, the southern North Sea (SNS). Locations of time-series stations are marked as circles.

Focal domain of this study is the southern North Sea (SNS) with open boundaries to the West and North, and water depths rarely exceeding 50m (Fig 1). Within the MOSSCO implementation, hydrodynamics is described by the General Estuarine Transport Model (GETM). Previous GETM applications extend from small scale coastal setups such as for the Wadden Sea [39, 40] to basin-wide simulations [41]. The curvilinear GETM grid for the SNS comprises 20 terrain-following layers and a horizontal resolution ranging from 1.5 to 5km. Further details of the set-up including physical forcing as well as validation studies of GETM for the SNS are given in [33, 32, 34]. Waves are represented by a simplistic statistical module. Different to relatively short-term coupled simulations [32], the dynamics of suspended particulate matter (SPM) is not explicitly tracked, but parametrized using a time- and depth-dependent function (see next but one subsection).

### Model for Adaptive Ecosystems in Coastal Seas (MAECS)

**Fig 2:**
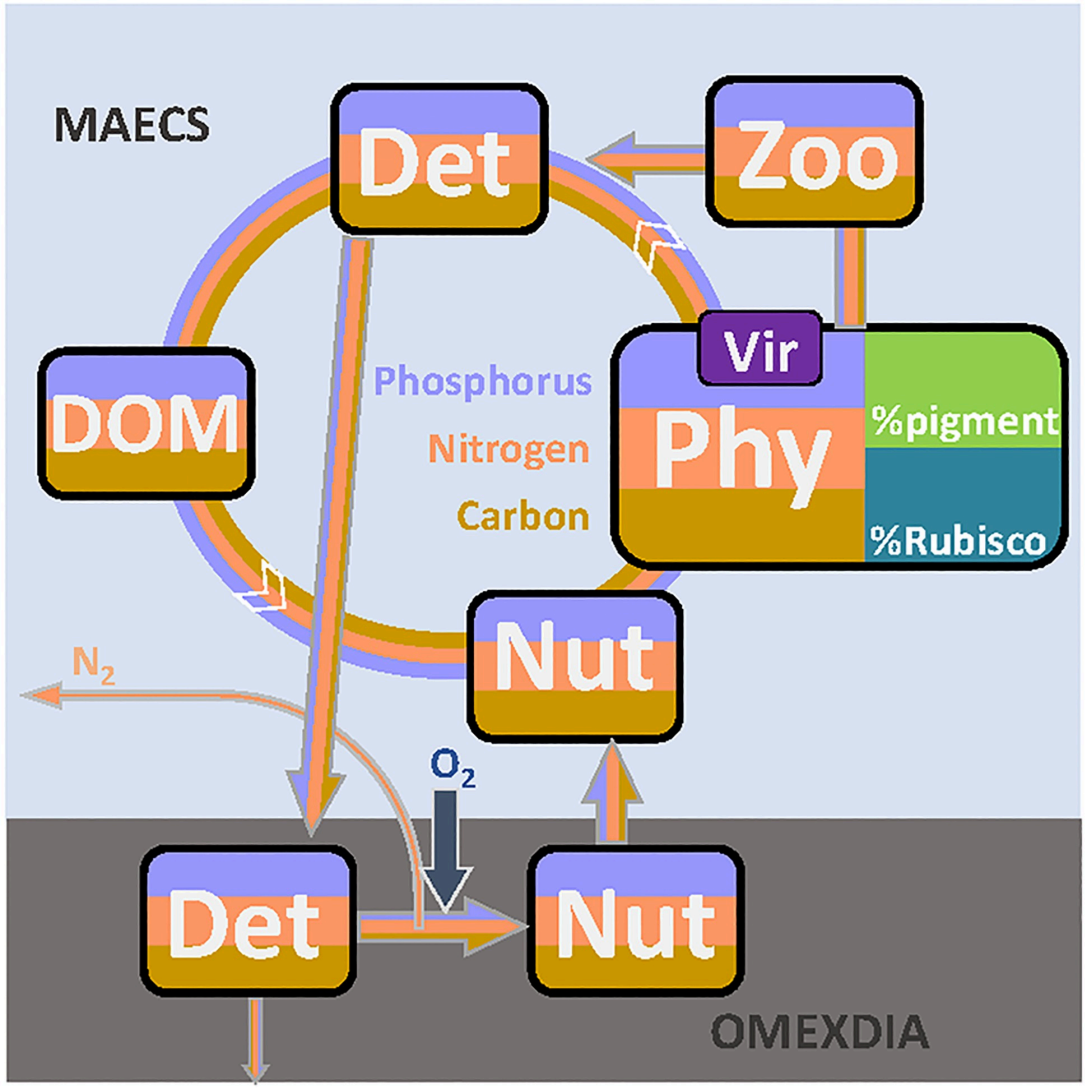
Major element flows described by the models MAECS (pelagic biogeochemistry) and OMEXDIA-P (benthic biogeochemistry, see Text A in S1 Appendix). The microbial loop of the three elements carbon (C), nitrogen (N), and phosphorus (P) starts with primary production by phytoplankton (‘Phy’). Its growth and mortality rates especially depend on physiological traits (here simplified by the two protein fractions invested into intracellular pigments and carboxylation machinery) and on virus leading to lysis. The microbial cycle continues to non-living particulate organic matter (‘Det’), dissolved organic matter (‘DOM’), and dissolved inorganic nutrients (‘Nut’). Part of the secondary production is channeled through a grazer compartment (‘Zoo’). Exudation from ‘Zoo’ and ‘Phy’ to ‘DOM’ is omitted in this graph. Settled organic material drives the benthic remineralization from ‘Det’ to ‘Nut’, which induces a net influx of oxygen and additional loss of elementary N through denitrification. A very small fraction of the benthic OM is buried, while the reflux of nutrients supports the pelagic cycle.

Pelagic ecosystem dynamics is simulated by the Model for Adaptive Ecosystems in Coastal Seas (MAECS), which is built upon a trait-based, physiological phytoplankton model [35, 33]. MAECS can resolve an arbitrary number of co-limiting elements relevant for photoautotrophic activity, as tested for silicate and iron, but has in our application been limited to carbon (C), nitrogen (N), and phosphorus (P). Uptake of these elements during phytoplankton growth, material flows of herbivorous grazing, and the turnover of detritus and dissolved organic matter in terms of C, N, and P are displayed in Fig 2. The pelagic biogeochemistry represented by MAECS is coupled within MOSSCO to the benthic diagenesis model OMEXDIA [42], which has been extended by few processes such as P-turnover as documented in Text A in S1 Appendix.

MAECS is one of the first models that explicitly tracks adaptive shifts in phytoplankton ecophysiology in a three-dimensional context. The underlying scheme for these adaptive shifts has been derived as an optimality theory and applied to phytoplankton growth and succession [43]. A first application of MAECS to the SNS including an analysis of plasticity effects has been presented by Kerimoglu et al., who provided a detailed description of pelagic biogeochemistry including turnover of organic and inorganic matter, phytoplankton losses due to aggregation and the variable sinking behavior of phytoplankton dependent on the physiological state [33]. Compared to that model version, the variant introduced in this study differs with respect to the account of spatio-temporal variations in (i) light attenuation, (ii) carnivorous grazing, and (iii) phytoplankton mortality by virus or parasites. The FORTRAN/FABM code of this model version is available at https://doi.org/10.5281/zenodo.2527733.

### Seasonal and lateral gradients in light attenuation

In high energy near-coast areas and after storm events throughout the relatively shallow SNS, large amounts of lithogenic and organic particles are eroded into the water column, leading to an increase of suspended particulate matter (SPM) up to the surface. This phenomenon is here captured by a non-linear dependency of SPM-related water attenuation *a*_SPM_ on few environmental variables: water depth *H*, turbulent kinetic energy dissipation *ϵ*_*b*_ at the bottom, and Julian day *J*,

$$\begin{array}{c} a_{\text{SPM}}=a_{\text{SPM}}^{\prime }\cdot [\alpha +\frac{1-\alpha }{2}\cdot (\eta_{20}\left(J\right)\;\sigma_{0.4}\left(H-H^{*}\right)+\sqrt{\frac{\epsilon_{b}}{\epsilon^{*}}}\;)] \end{array}$$ (1)

where $a_{\text{SPM}}^{\prime }$ denotes a specific attenuation coefficient, *α* a constant relative background contribution of SPM or other optically active constituents such as colored dissolved organics matter (CDOM), H* the threshold water depth for resuspension, and ϵ* the critical bottom turbulent kinetic energy dissipation rate for resuspension (see the list of newly introduced MAECS coefficients in Table A in S1 Appendix). The functional form of *a*_SPM_(*H*, *J*, *ϵ*) is illustrated in Fig A in S1 Appendix. Its parameterization reflects a previous analysis of the SCANFISH turbidity data for the eastern part of the SNS [44]. The auxiliary periodic function

$$\begin{array}{c} \eta_{L}\left(J\right)=\frac{1}{2}+\frac{1}{2}\cos(\frac{2\pi }{365d}\;\left(J-L\right)) \end{array}$$ (2)

by construction reaches an annual maximum at day *J* = *L* (here 20d). The sigmoid function *σ*_*s*_(*x*) introduces the steepness parameter *s*:

$$\begin{array}{c} \sigma_{s}\left(x\right)=\frac{1}{1+\;e^{s\cdot x}} \end{array}$$ (3)

Taken together, the first term in the large round bracket of Eq (1) can be interpreted as the average turbidity distribution that peaks in winter and shallow areas (*H*<20m), while the second one gives a moderate contribution at the event scale, since energy dissipation at the bottom varies with tides and during storms, also as a result of wave action [30].

### Parametrization of herbivorous and carnivorous grazing

The dynamics of herbivorous grazers in MAECS is in detail explained in [33], but here modified with respect to the half-saturation coefficient and the mortality by carnivorous grazing. For physical and ecological reasons, both characteristics are assumed to alter in the vicinity to the shallow parts of the SNS, namely the Wadden Sea and estuaries. This vicinity is quantified not unlike the cross-shore turbidity function in Eq (1), also re-using some of its coefficients such as slope and transition depth. The auxiliary variable ${\tilde{\sigma }}_{H,S}$ combines three sigmoid functions *σ*_*s*_ (defined in Eq (3)), one depending on water depth *H* and two others on salinity *S* (in PSU):

$$\begin{array}{c} {\tilde{\sigma }}_{H,S}=\max\{\sigma_{0.4}\left(H-H^{*}\right),\sigma_{1}\left(S-31\right)\}+\frac{1}{2}\sigma_{1}\left(S-12\right) \end{array}$$ (4)

In addition to the purely bathymetric water mass characterization Eq (1), the two salinity thresholds further refine the transition from open shelf areas to two water bodies, typical coastal water with PSU around or below 31, and to semi-haline water at PSU below 12 close to estuaries. Both salinity-based formulations introduce variability due to displacements of salinity fronts in the SNS as observed [45] or simulated by GETM [41, 33].

Zooplankton behavior is known to differ between coastal and offshore waters. For example, copepods reveal significant vertical migration and accumulation only in offshore areas [46]. As a consequence, zooplankton will much better exploit vertically non-uniform prey densities in deeper waters, which are a typical feature of phytoplankton profiles simulated by MAECS for the mixed-stratified SNS [4, 33]. In the ocean, spatial or vertical aggregations of phytoplankton are often accompanied by much increased densities of grazers [47, 48]. Vertical migration thus enhances feeding efficiency and affinity, which is here implicitly captured by multiplying the half saturation constant for herbivorous grazing by a factor of $1/2+{\tilde{\sigma }}_{H,S}$. In coastal waters with higher ${\tilde{\sigma }}_{H,S}$, saturating conditions demand higher prey concentrations compared to offshore areas.

Mortality rates of herbivorous grazers can be expected to increase in shallow waters due to ecological factors such as predation and also entail a strong seasonal signal. Maar et al. reconstructed the spatio-temporal distribution of biomass specific mortality of mesozooplankton due to fish (*m*_Z_) using trawl surveys for the entire North Sea [21]. They found that *m*_Z_ reaches maximal values in nearshore areas of the English coast and the Wadden Sea and along an offshore transect from the Dogger Bank to the Northern Danish coast, thus at or beyond the border of the SNS setup. Apart of the plume region of the Dutch and Belgian rivers at the southwestern part of the SNS, the distribution is matched by our simple vicinity index ${\tilde{\sigma }}_{H,S}$. In addition, Maar et al. found a very strong seasonality in *m*_Z_, about one to two orders of magnitude difference from low winter values to the summer peak. This empirical finding is here described by the periodic function already defined for SPM-seasonality, *η*_20_(*J*) in Eq (1), which is phase shifted by slightly more than half a year, made more pronounced by taking the square ($\eta_{250}^{2}\left(J\right)$) and amplified by a specific factor *β*. The overall specific mortality including a temperature dependence *f*_*T*_ for the activity of top-down predation then reads

$$\begin{array}{c} m_{Z}=m_{Z}^{\prime }\cdot f_{T}\cdot {\tilde{\sigma }}_{H,S}\cdot \left(\gamma \;Z+\beta \;\eta_{250}^{2}\left(J\right)\right) \end{array}$$ (5)

and is visualized as a function of water depth *H* for different days in the vegetation period in Fig A in S1 Appendix. Superimposed on the average seasonality is a variable contribution at the event scale (see *a*_SPM_ in Eq (1)), here expressed by a quadratic mortality term *γZ* where *Z* denotes the zooplankton concentration. This term also accounts for non-predation mortality such as arising from diseases. Because of the additional contribution of the density dependence and the seasonal elevation during summer, the mortality amplitude $m_{\text{Z}}^{\prime }=0.025{\text{d}}^{-1}$ is lower than the maximal values around 0.04d^−1^ suggested by Maar et al. [21]. The rationale for a carnivory function similar to Eq (5) comprises various aspects of coastal ecology. Turbidity, with the spatio-temporal distribution estimated above, has been reported to limit feeding of fish [49, 50, 51]. This limitation will apply more to the larger fish with greater search volume. As a consequence, predation risk declines for smaller organisms, and fish juveniles indeed generally prefer (coastal) turbid waters [52, 53]. These small fish size classes make the predominant predators of zooplankton so that specific zooplankton mortality *m*_Z_ will increase with rising turbidity or decreasing distance to the coast, respectively. Salinity intolerance as parametrized through *σ*_1_(*S* − 31) and *σ*_1_(*S* − 12) in Eq (4) will affect a part of the zooplankton community in vicinity of large estuaries. A high net mortality rate in the zooplankton community of 0.05 d^−1^ were reported for the Westerschelde estuary [54]. Not only young fish but also mussels ingest higher numbers of copepods—with a preference towards the smaller size classes [55, 56, 57]. A joint model–data study indicated that this size selectivity vanishes under enhanced turbulence levels as typical for the Wadden Sea and that loss rates of copepodites above mussel beds may range from 0.07 to 10 d^−1^ [58]. As a result, benthic filter feeders can significantly contribute to enhanced near-coast pelagic carnivory, with occasionally extreme removal rates. If we assume carnivory outside the mid-summer peak to derive by about one third from benthic suspension feeding, the model parametrization in Table A in S1 Appendix is equivalent to mortality rates by mussels below 0.1d^−1^, which is compatible with the lower range of those estimates.

### Viral dynamics and infection

Infecting symbionts or pathogens such as a lytic virus are in the new version of MAECS described by the intracellular viral density *v*. Different to other virus models, this variable is normalized so that *v* = 1 corresponds to the lethal dose (”LD50”) where half of the host population dies. Host mortality *m*_P,vir_ is formulated as a smooth step function of *v* (see Eq (3)),

$$\begin{array}{c} m_{P,\text{vir}}=1d^{-1}\cdot \sigma_{s}\left(1-v\right) \end{array}$$ (6)

By definition, the proportionality parameter equals 1d^−1^ such that a 50% loss per day happens at critical pathogenic density *v* = 1 due to *σ*_*s*_(1 − *v*) = *σ*_*s*_(0) = 1/2. Lytic viruses ultimately kill their host once the burst size is reached, which is here expressed by a relatively steep transition (*s* = 4). Already at *v* lower than one the intracellular virions damage the metabolism of the host, while at massive viral infection the deleterious impact will not furthermore scale with the viral densities, an observation often made in diseases and formulated in tolerance–saturation–models [59]. However, due to intense viral removal processes, densities will rarely exceed *v* = 1, even during longer lasting outbreaks such that Eq (6) will impose mortality rates of in the order of 0.2d^−1^, which is compliant to scarce field observations [60, 61]

In terms of biogeochemical fluxes within MAECS, viral mortality *m*_P,vir_ of autotrophic biomass dominantly fuels the detritus pool (80%), while the remainder is passed to the dissolved organic pool. Viral density *v* is assumed to change due to three aggregate processes, (1) infection–replication, expressed by the rate of viral adsorption at the host, *p*_ads_ and subsequent multiplication by replication *n*_rep_, (2) virus removal by selective host dynamics *r*_defense_, also termed antiviral defense, and (3) virus mortality *r*_mort_:

$$\begin{array}{c} \frac{d}{dt}v=r_{\text{ads}}\cdot n_{\text{rep}}-r_{\text{defense}}-r_{\text{mort}} \end{array}$$ (7)

Dependencies of the ruling rate functions on ambient and intracellular conditions are derived in Text B in S1 Appendix.

Although eukaryotic phytoplankton are predominantly infected by lytic viruses that destroy their hosts upon burst of replicated virions, the virulence intensity *v* is here formulated as an intracellular component, thus mimicking a lysogenic pathogen. This way, extreme and artificial host-virus ratios were avoided even in unconstrained 3D simulations. As internal variable associated to phytoplankton biomass concentration, *v* is transported in the three-dimensional ocean alike the MAECS trait variables.

### Data integration

The reference hindcast simulation was run from 2000 to 2014, leading to a sufficient overlap with the selected monitoring data. These data represent characteristic lateral and seasonal gradients in major ecosystem states from the near-cost, mostly the Wadden Sea and major estuaries, to transitional waters of the SNS. Biogeochemical validation data derived from an ensemble of Dutch and German time-series stations: Dissolved inorganic nitrogen (DIN), dissolved inorganic phosphorus (DIP), and CHL measurements at the two transects Noordwijk and Terschelling, from which two stations each were taken (NORDWK10, NORDWK70, TERSLG4, TERSLG50, numbers denoting the distance to the shore). These time-series can be directly downloaded from the WATERBASE data collection of Rijkswaterstaat hosted at http://opendap.deltares.nl. Other monitoring data for CHL, DIN, and DIP were made available by German institutes and authorities: for Sylt from AWI (Alfred-Wegener Institute, van Beusekom, pers.comm., [62]), Norderelbe from LLUR (Landesamt für Landwirtschaft, Umwelt und ländliche Räume des Landes Schleswig-Holstein, Petenati, pers.comm.), and Norderney from NLWKN (Niedersächsisches Landesamt für Wasserbau und Küstenschutz, Grage, pers.comm.). Mesozooplankton abundance data from the Helgoland Roads monitoring station available until the end of 2004 (W. Greve, pers. comm., and [63]) were converted to biomass similarly to the procedure described in [21], but with different specific body weights and selection of key species/groups as specified in Table B in S1 Appendix. Remote sensing fluorescence CHL data are available from the European Space Agency, Ocean Color—Climate Change Initiative (ESACCI) version 3.1, at http://www.esa-oceancolour-cci.org. The product reaches high coverage through a compilation of the three sensors MERIS, MODIS and SeaWiFS, and all scenes with a coverage of more than 30% of the SNS were selected. Fluorescence CHL data were generated using advanced processing algorithms [7]. Nonetheless, the ESACCI product considerably deviates from the *in situ* data of the Dutch and German monitoring stations and is therefore further transformed as documented in Fig B in S1 Appendix.

Atmospheric boundary conditions from CLM (Climate Limited-area Modelling) re-analyis data are part of the COASTDAT repository (www.coastdat.de). Pelagic boundary conditions for DIN and DIP along the North Sea and east of Dover Strait originated from ECOHAM, zooplankton biomass was set to 0.2 mol-C m^−3^ and phytoplankton biomass assumed to be negatively correlated to inorganic nitrogen (7mol-C m^−3^-DIN/3 mol-C mol-N^−1^). Riverine freshwater and nutrient discharge were assembled at daily resolution and both interpolated and extrapolated using trend analysis and climatological seasonal cycles. The synthesized data set including the various sources can be found at www.mossco.de/rivers. Riverine N and P loads were then multiplied with a global retention factor of 0.85, which is a conservative average for few estimates of recent estuarine nutrient retention in continental rivers ranging from 5–10% [64] to 20% [65] for the Elbe estuary or 20%–30% for the Scheldt estuary [66]. Atmospheric deposition of N was compiled as long-term constant climatological field from EMEP reconstructions [67], however elevated by a factor of three in order to reach typical values estimated for the SNS [68]. Deposition of P was set 0.01 mol-P/mol-N times the N flux [69].

### Numerical experiments

Benthic and pelagic states were brought close to an equilibrium cycle by an initial spin-up of 15 years. Model results were stored at 1,5d intervals and, after log-transformation, compared with the observations both visually and using standard error statistics. For the time-series comparison of station data, the best matching value within a 3d time window at each measurement event were determined to reduce mismatches due to phase lags within day-night and tidal cycles between simulated and observed data. In addition to the reference run I conducted numerical experiments for unraveling how the two introduced features, viral dynamics and non-uniform carnivory affect the simulation results. For the “No Virus” scenario, the replication rate *n*_rep_ in Eq (6) was set to zero inducing a fast disappearance of pathogens. The second scenario “Uniform Carnivory” was more difficult to establish since the seasonality in the zooplankton mortality rate *m*_Z_ given Eq (5) should be preserved. Lateral gradients were annihilated by keeping water depth and salinity in Eq (4) constant at *H* = H* and *S* = 31PSU, which characterizes both an average and transitional SNS water body. Hence, the spatial average of *m*_Z_ was roughly conserved compared to the reference run.

## Results

### Cross-shore nutrient gradients

**Fig 3:**
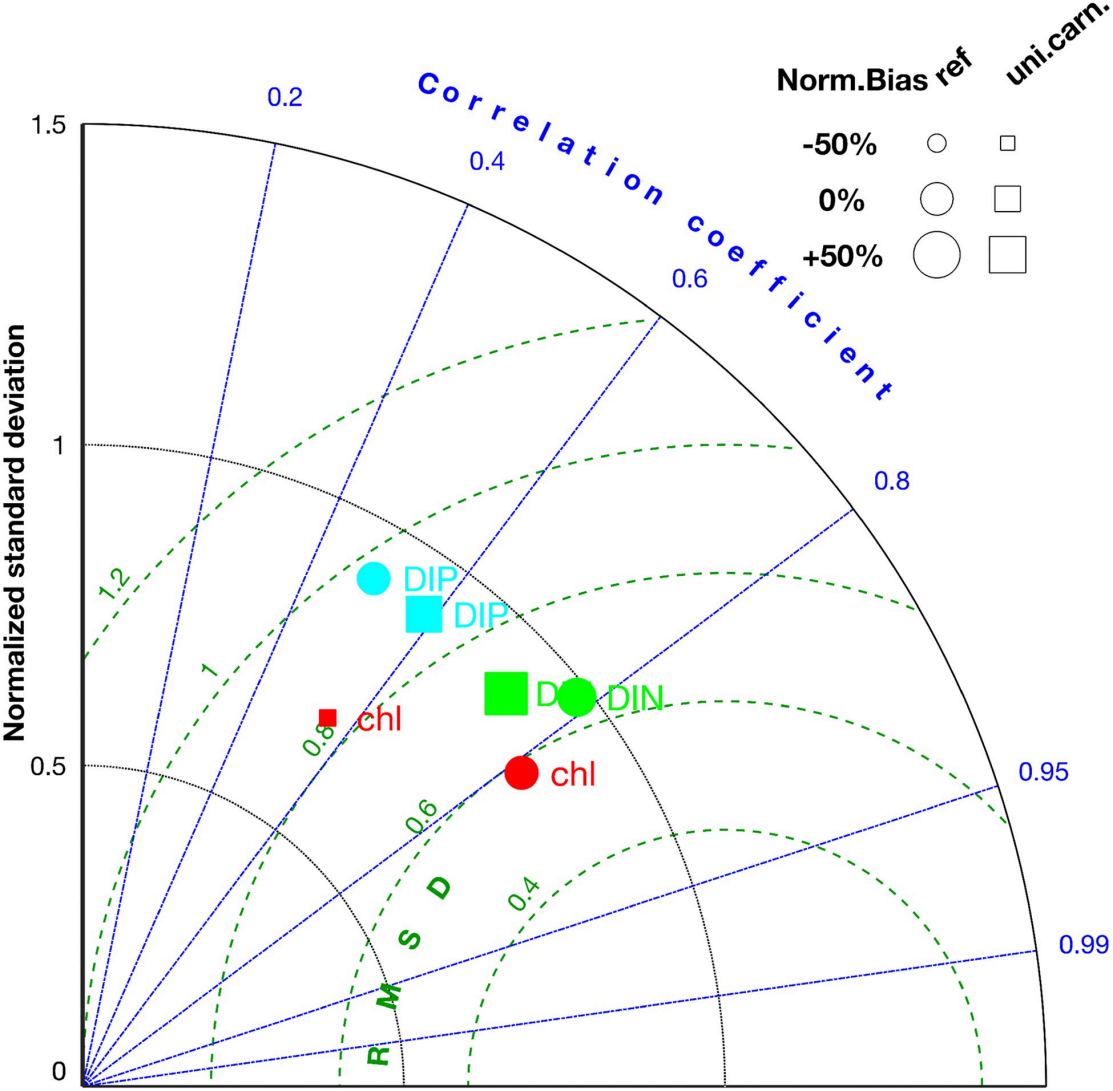
Taylor diagram displaying four statistical measures of the comparison of model results with station time-series data. (1) The mean-normalized bias determines the size of the plot symbols, (2) the root-mean-squared deviation (RMSD) normalized by the standard deviation in the data, STD, the distance from the reference point at the right bottom, (3) the STD-normalized standard deviation in the simulated time-series the radius position in the non-linear polar grid (black curves), and (4) the correlation between model and data the angle (blue lines). The measures of the reference run (circles) and the scenario of “uniform carnivory” (squares) are compared for the three observables DIN (green), DIP (cyan), and CHL (red).

Nutrient concentrations calculated by MAECS in average exhibit a steep cross-shore gradient, which however diminishes during summer, especially for DIP. The gradient and its smoothing during summer are evident in the climatological (2000-2014) maps in Fig C in S1 Appendix and by comparing long-term time-series between the near-coast stations (Sylt, Norderelbe, Norderney, Terschelling-4, Noordwijk-10) and the more offshore stations (upper diagrams in Figs D and E in S1 Appendix: Noordwijk-70, Terschelling-50). Winter DIN values at the near-coast stations range from 50 to 300 mmol-N m^−3^ in contrast to 5 to 40 mmol-N m^−3^ at the offshore stations. In both seasons, DIP varies cross-shore by a factor of 2-8, which is much less compared to the gradient in DIN that covers two orders of magnitude over a short distance. These patterns of the reference simulation are supported by the compiled measurements. Strong P-depletion builds up near-shore during early summer, accompanied and followed by N-depletion in all shallow areas not adjacent to a major estuary as displayed in Fig C in S1 Appendix. The succession of P– and N–depletion is confirmed by the monitoring data (Figs D and E in S1 Appendix), although summer DIP is underestimated by the model at Norderelbe and overestimated at Noordwijk-10. Notwithstanding the seasonal variations of two orders of magnitude, many singular events or fluctuation amplitudes at different stations agree very well, in some cases even with high accuracy. Taken together, the model displays a sufficient to high skill in reproducing multi-scale temporal and spatial variability in coastal inorganic nutrients, as also quantified by the error statistics of the Taylor diagram in Fig 3. For DIN, and in comparison to the predecessor version of MAECS [33], inferior errors appear as rather small root-mean-squared deviation, RMSD, (63% of the standard deviation in the data, STD), a relatively high correlation of around 0.8, and excellent first order statistics expressed by 18% bias and nearly the identical STD in the reference simulation and the data. The skill for DIP moderately lags behind, which is to a considerable degree due to the underestimated summer DIP at Norderelbe (leaving aside this station shifts the DIP error statistics close to that for DIN, not shown).

### Multi-scale variability of chlorophyll

**Fig 4:**
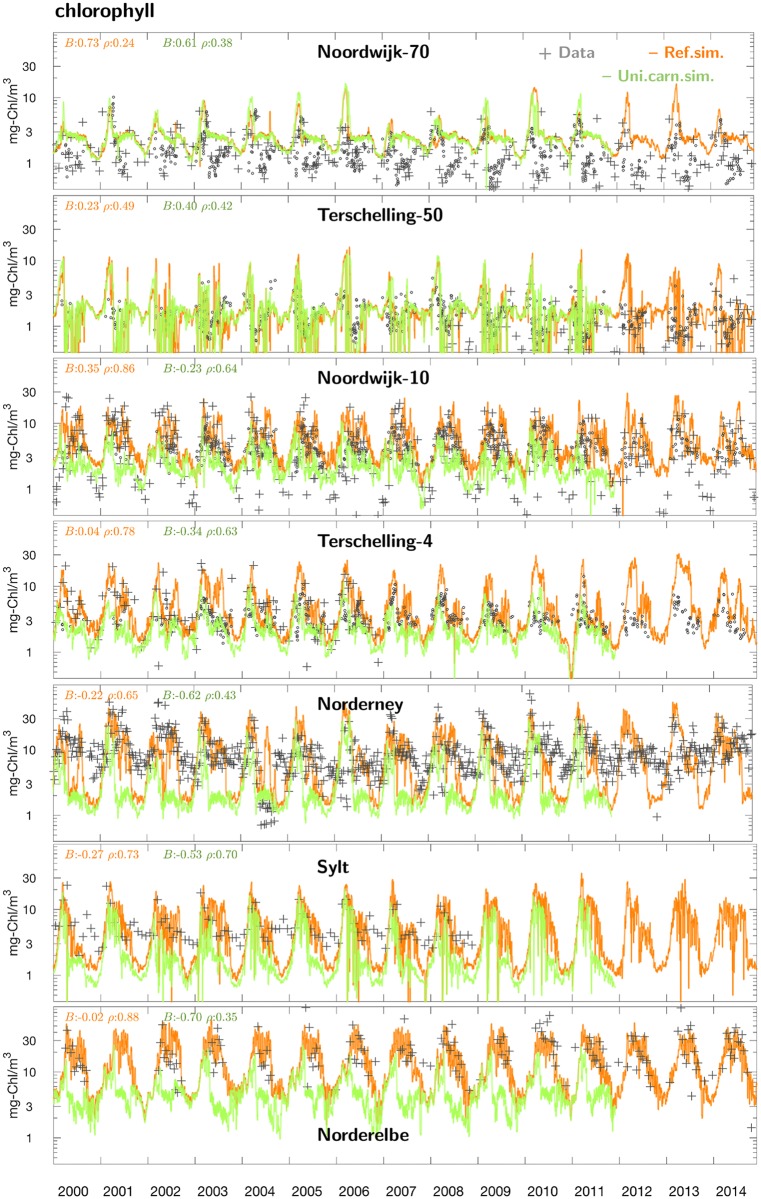
Long-term dynamics of at 7 stations in the southern North Sea. The stations are ordered according to the distance to the coast with the most offshore station (Noordwijk-70) at the top. Compiled measurements (gray crosses) are compared to the reference run (orange line) and the run with spatially uniform carnivory (green line). This scenario is only plotted until end of 2011 for better visibility. Normalized bias *B* and correlation coefficient *ρ* of the model–data comparison are added in the color of the respective scenario.

Typical simulated CHL concentrations during spring scatter around 4–10 mg-Chl m^−3^ at the offshore stations and around 15–50 mg-Chl m^−3^ near-shore, thus again underlining a pronounced cross-shore gradient (Fig 4). Similar to nutrients, seasonal fluctuations span more than one order of magnitude. At all stations apart of Norderelbe, a clear water phase emerges that separates the spring bloom from one or more summer blooms, both in the time-series data and the simulations except for Noordwijk-70. While the observed CHL peak values are in general very well captured by MAECS in terms of magnitude and timing, autumn and winter values are generally overestimated at both Noordwijk stations and underrated at Norderney and Sylt. Winter observations are sparse at Norderelbe but the extreme CHL concentrations of about 50 mg-Chl m^−3^ are well fitted, akin to the 500 times lower winter and early summer values at the offshore station Terschelling-50. The overall high skill of the model to reproduce large temporal fluctuations across different sites is approved by the error statistics. A model-data correlation above 0.8 for CHL exceeds even the one for DIN, but also normalized RMSD (below 0.6) and normalized standard deviation (0.8) can be regarded as very good for reproducing a biological observable.

**Fig 5:**
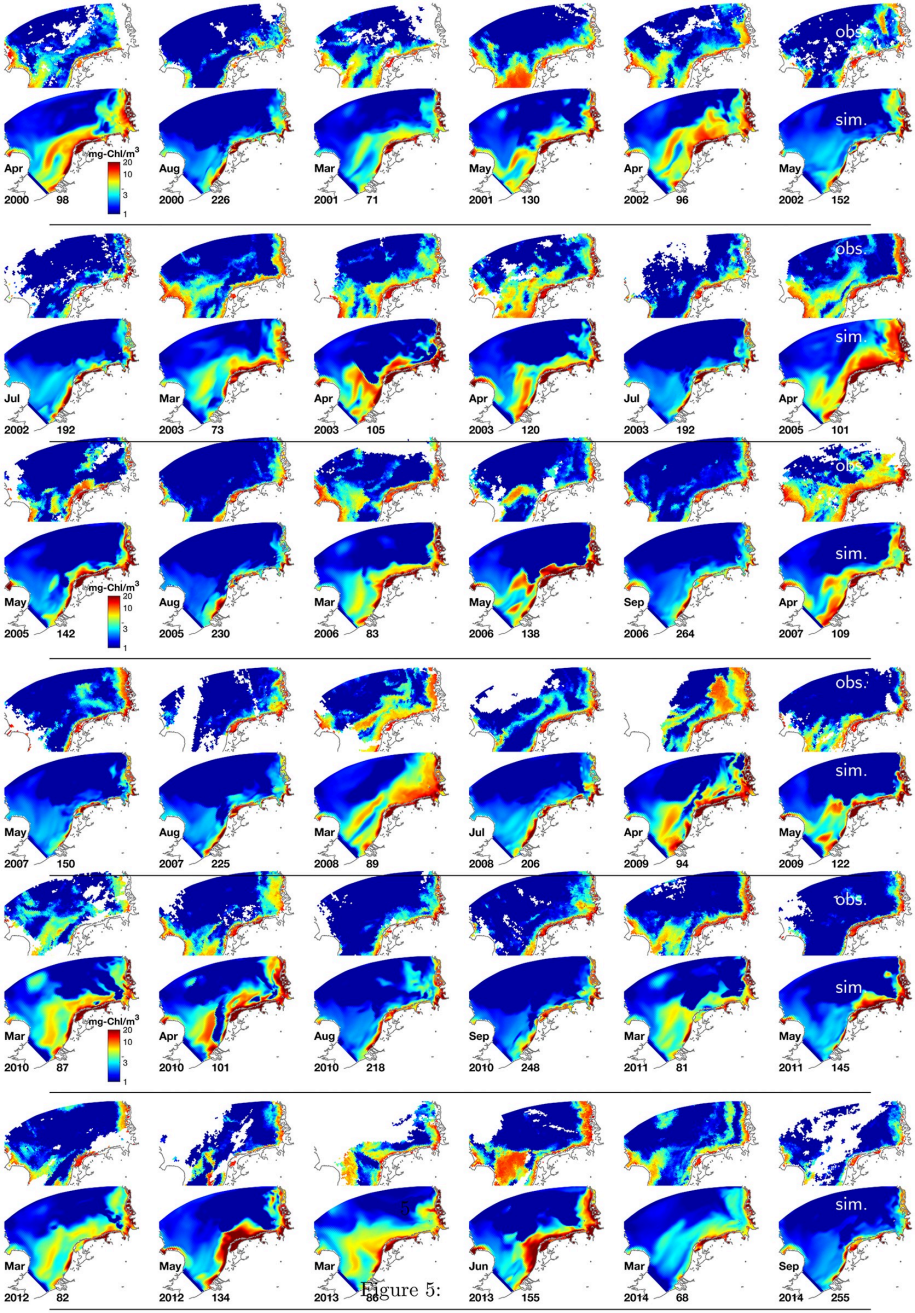
Snapshots of surface CHL from 2000 to 2014 at different Julian days in the southern North Sea, simulated (even rows) and observed (ESACCI, odd rows).

**Fig 6:**
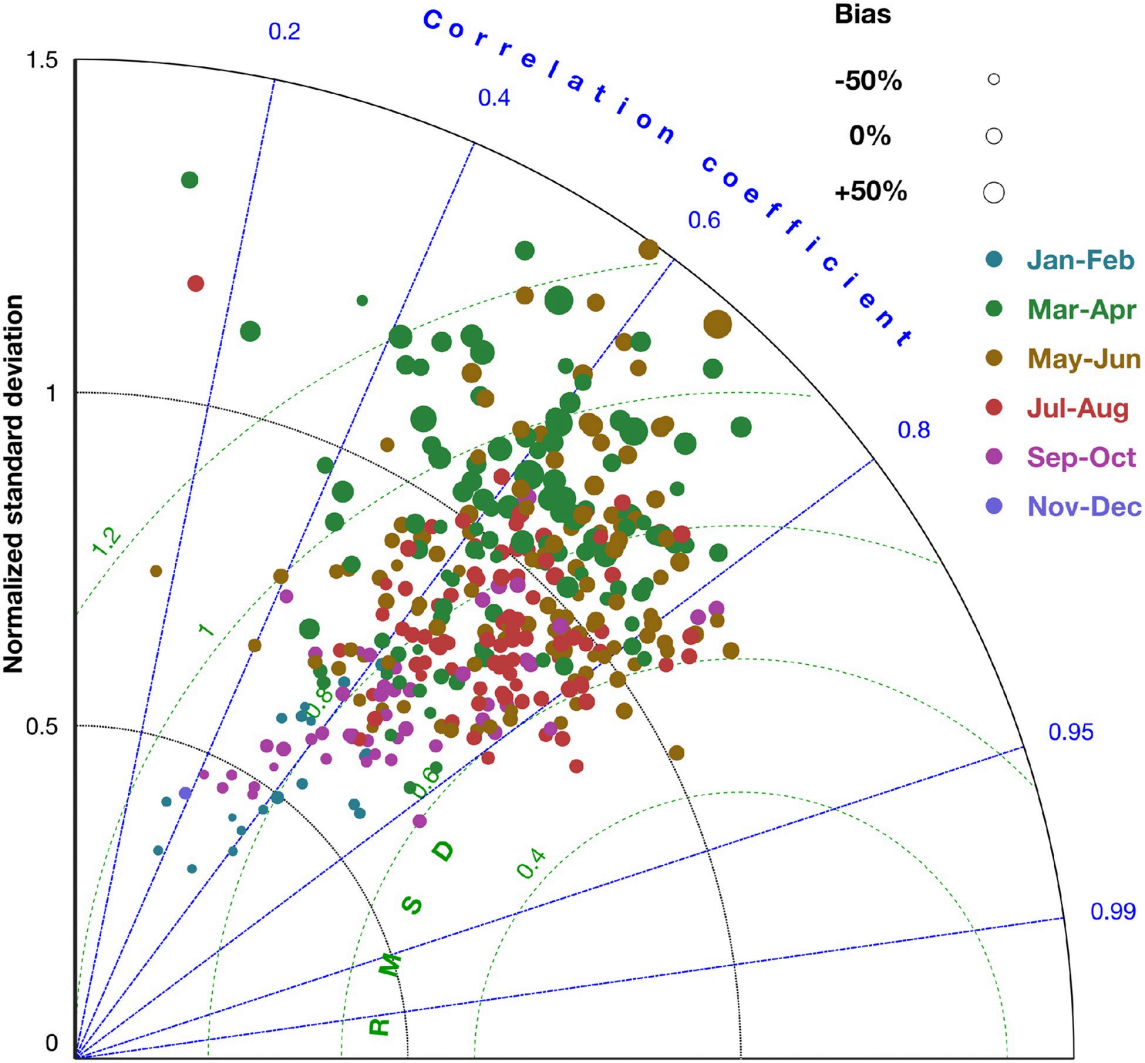
Temporal sequence of surface chlorophyll-a in 2004 for the three scenarios compared to observations. Maps derived from remote sensing (ESACCI, top row), and from three different simulations, the simulation lacking virus dynamics (2nd row), the reference run (3rd row), and the run with spatially uniform carnivory (4th row).

**Fig 7:**
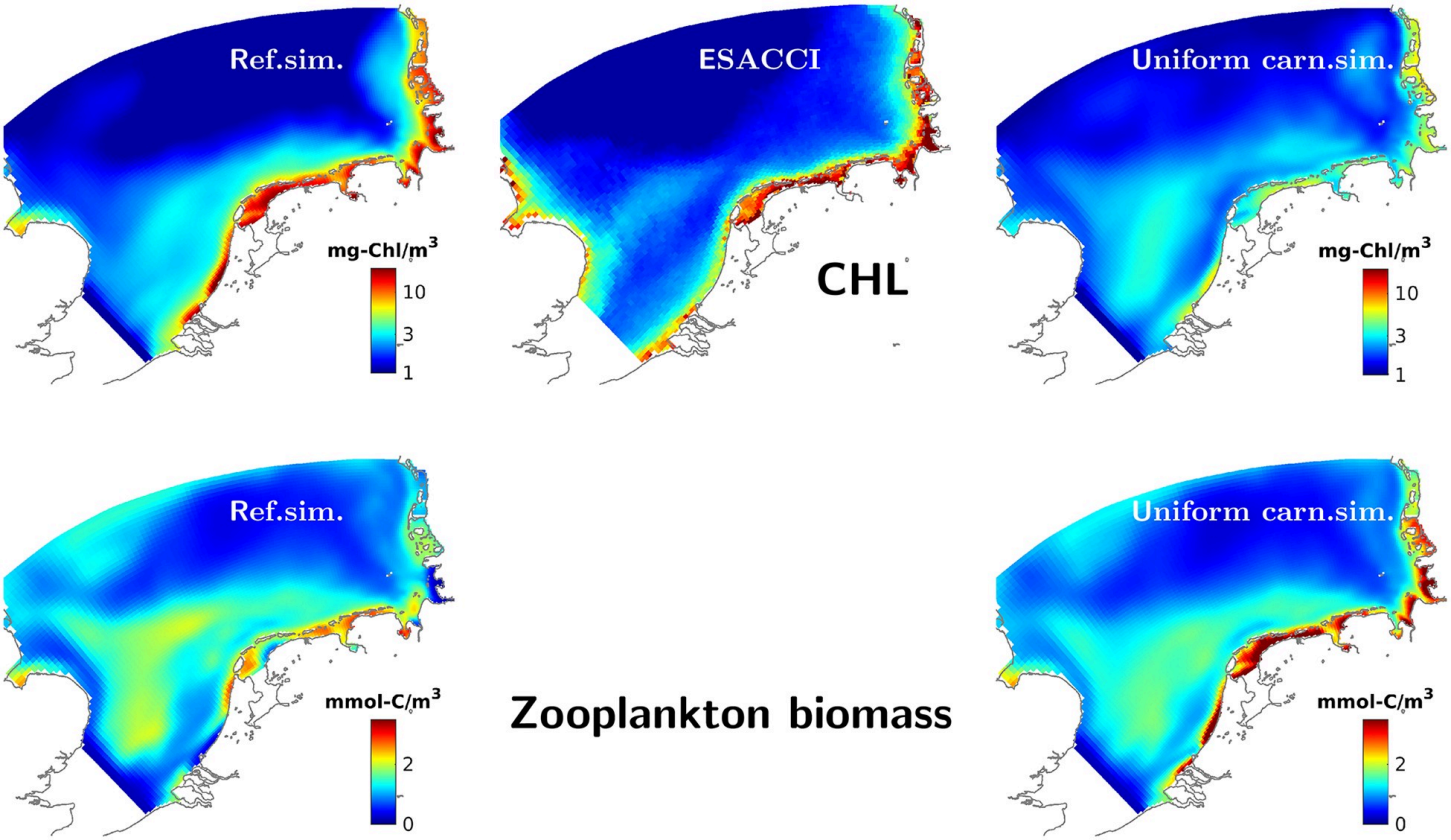
Taylor diagram displaying model error statistics with respect to remote sensing CHL data. The mean-normalized bias (symbol size), the root-mean-squared deviation normalized by the STD of the data (green iso-lines), the STD-normalized standard deviation in the simulated CHL map (black isolines), and the correlation (blue angles) are shown for the reference run where comparisons using individual scenes within the period 2000-2014 are seasonally pooled (color code).

A sequence of snapshots for typical spring and summer surface CHL distributions in Fig 5 confirms the similarity between the simulated and remotely observed (ESACCI) maps. Both in the data and the reference run the strong cross-shore gradient in CHL persists throughout all years. The spring bloom disperses into offshore areas whereas thereafter most surface layers at water column depth above 20m remain clear (below 1-2 mg-Chl m^−3^). The frequent jet-like feature called the East Anglia Plume, carrying relatively high CHL concentration from the eastern UK coast parallel to the continental coastline through the entire western SNS, is often matched by the model albeit moderately transposed to the south. Other concurrent features in satellite images and MAECS include the strong patchiness especially in the transitional zone, generated by intermittent CHL structures at the scale of 5–50km, or a recurrent band of reduced CHL parallel to the North Frisian coast (2002/152, 2009/94, 2014/68 in Fig 5). In summary, single intermittent blooms may be missed by the model, but the overall distribution of simulated band- and patch-like meso-scale structures and the degree of interannual variability in those structures is compliant to the data. Even smaller coastal blooms are again fitted in terms of shape, location and intensity, shown also at higher temporal coverage in Fig 6 (e.g., day 85 or 221), and so is lateral variability as such, in terms of typical extension and life-time of surface CHL structures. The Taylor diagram for all 381 individual scenes reflects a generally high skill in reproducing spatial patterns, which however differs between seasons (Fig 7). For spring and early summer, MAECS often predicts slightly too high absolute values (positive bias) and normalized STD, while the opposite is the rule for late autumn and winter situations. During summer, most lateral patterns in 2000-2014 are redrawn with a bias below 25%, normalized RMSD of 0.6-0.8, and correlation coefficient again between 0.62 and 0.84, thus close but somehow inferior to the skill statistics based on the station data. The small spread of roughly normal distributed correlation coefficients indicates a systematic capability of the model to capture variable summer CHL patterns (Fig G in S1 Appendix).

**Fig 8:**
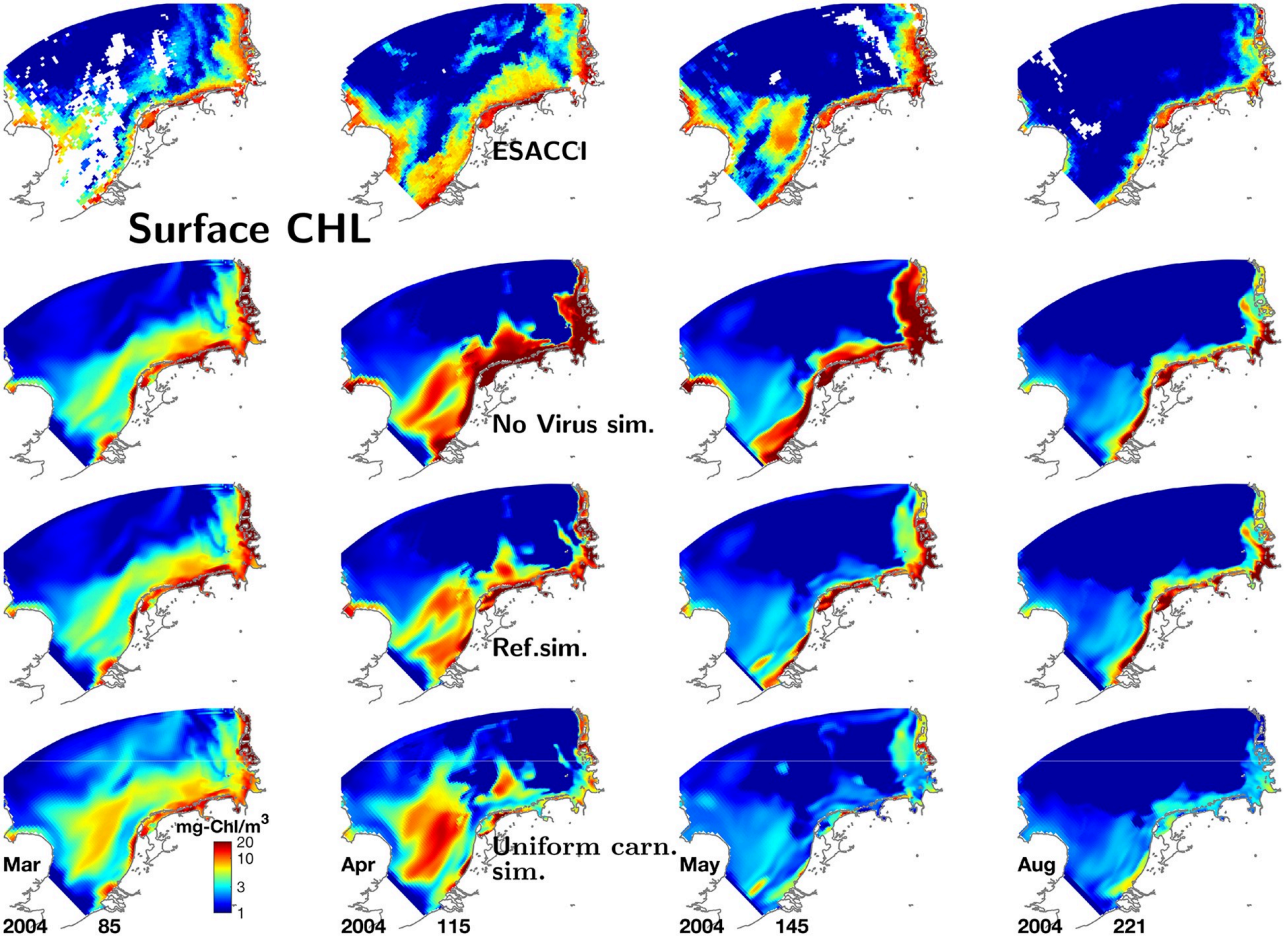
Climatological surface CHL and zooplankton. Top: Long-term (2000-2014) averaged result of the reference model (left panel) and of the version neglecting spatial gradients in carnivory (right) compared to satellite derived data (mid panel). Bottom: Long-term and vertical average of simulated zooplankton biomass for the reference and scenario run, respectively.

As expectable from the snapshop comparison and the error statistics for individual CHL scenes, the average spatial characteristics of the long-term (2000-2014) CHL distribution largely converge between satellite observations and model, both at the annual and seasonal scale (Fig 8 and Fig F in S1 Appendix). However, MAECS overestimates CHL in the transitional zone in the first half of the year (Dec-May) and underestimates the high values –extending also to offshore areas– observed in late autumn (Sep-Nov, Fig F in S1 Appendix and Fig 7). The model in particular predicts a too weak coastal CHL accumulation during winter (Dec-Feb). These discrepancies are inherent to the seasonally resolved skill statistics in Fig 7, but not very evident when referring to the *in situ* time-series data (Fig 4).

### Seasonal fluctuations in zooplankton and virus

**Fig 9:**
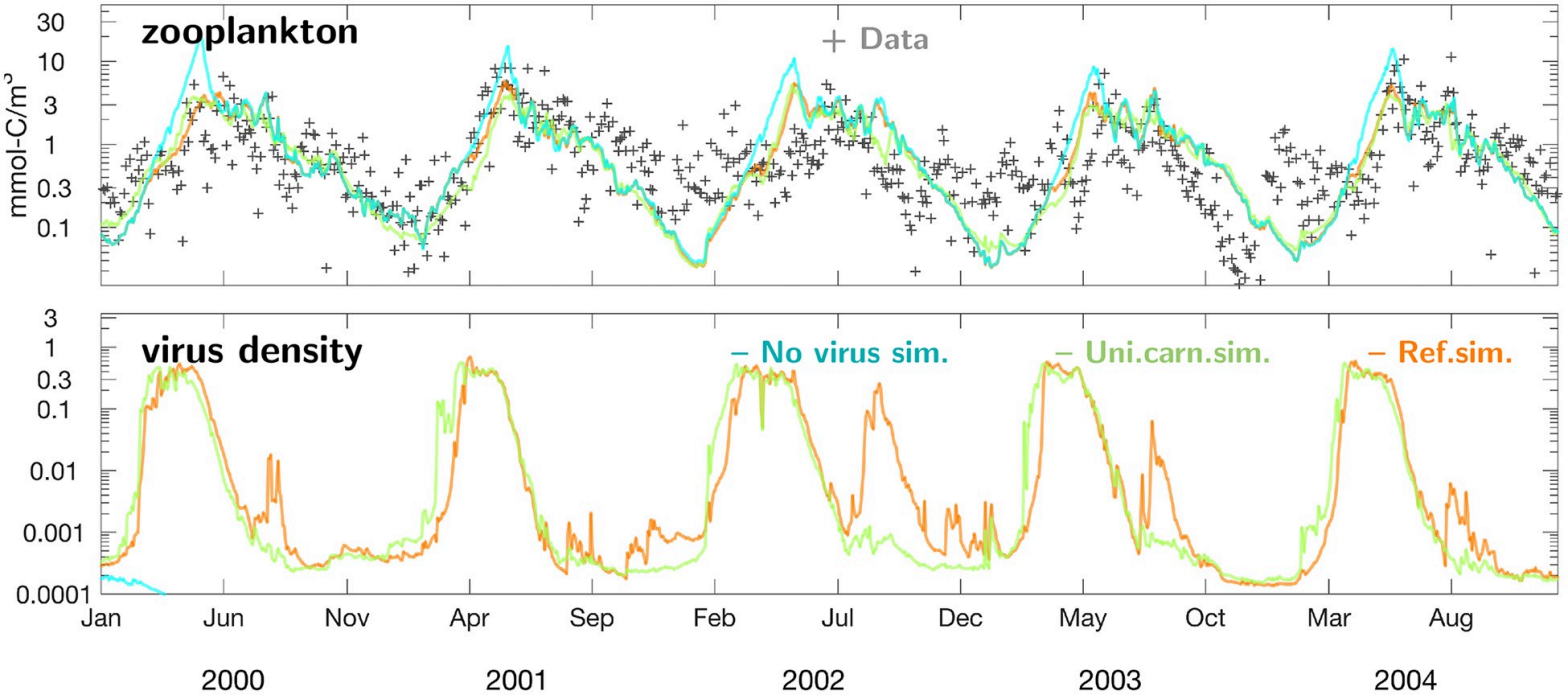
Factors of phytoplankton mortality. Top: Five annual cycles of simulated zooplankton biomass (lines) versus mesozooplankton biomass calculated using count measurements (black crosses) at Helgoland. Bottom: Simulated dynamics of intracellular virus density. Again results of three simulations are shown: reference run (orange), the run with spatially uniform carnivory (green), and the one lacking the virus compartment (cyan).

Zooplankton biomass measured at Helgoland peaks around June, with interannual differences of about one month (Fig 9). Model and data agree with respect to (i) average timing, (ii) the continuous rise in zooplankton biomass by two orders of magnitude over about three months, (iii) the slow and discontinuous decline thereafter, (iv) and returning higher concentrations in autumn. However, peak values are sometimes underestimated in the reference run, whereas the simulated zooplankton biomass shown in Fig H in S1 Appendix again matches or even moderately overestimates values from the Continuous Plankton Recorder (CPR) with summer peak values of 1.5–2 mmol-Cm^−3^ (CPR box C1), 1.6–3 (D1), and around 2 (D2) [21].

Virus density, also shown for Helgoland in Fig 9, displays a much more rapid and earlier bloom than herbivorous zooplankton, passing three orders of magnitude within few weeks. Thereafter, simulated viral infections in phytoplankton remain stable during about two months, after which they vanish abruptly. Only in 2002, when the summer CHL bloom exceeds the level of the spring bloom (Fig 4), the model predicts a second viral outburst.

### Effect of spatial uniformity in carnivory

A spatially uniform specific mortality rate of herbivorous zooplankton leads to changes across all ecosystem compartments. First, summer nutrient concentrations in near-shore areas turn out higher in that scenario (Figs D and E in S1 Appendix, and Fig 3). This increase goes parallel to altered phytoplankton dynamics (Fig 4): At all monitoring sites apart of the offshore stations Noordwijk-70 and Terschelling-50, CHL is much reduced throughout the year in the uniform carnivory scenario compared to the reference run, in particular during summer where the reduction often exceeds one order of magnitude, thus leading to large deviations from the measured CHL concentrations. These deviations are in a compact way represented by significant deleterious shifts in all four skill measures (Fig 3). Their spatial outlay is here presented for one example year (2004) in Fig 6: the spring bloom in April 2004 is stronger offshore in the scenario simulation compared to both the reference run and the satellite data. By contrast, the scenario yields much lower near-shore CHL values after day 100. The characteristic band of high CHL in the backbarrier reefs of the Wadden Sea islands hardly develops, which is a typical result during the entire simulation period as demonstrated by the climatological maps in Fig 8 and Fig F in S1 Appendix, in the latter differentiated according to season. In the simulation with uniform specific zooplankton mortality, the near-coast CHL accumulation undervalues the observed one by roughly one order of magnitude from late spring to autumn. Consequently, only a negligible fraction of scenes is better reproduced by the uniform carnivory scenario during summer, as evident for the correlation distribution (Fig G in S1 Appendix) but also for all other error measures (not shown).

While the reference run nearly perfectly fits the long-term averaged near-coast distribution and only moderately displaces large scale structures in the transitional zone, the variant with spatially uniform carnivory turns the steep cross-shore CHL gradient into a smooth one, predicting too high offshore surface CHL concentrations and by far too low ones close to the coast. This diminution of the CHL gradient is caused by the massive accumulation of herbivorous grazers predicted near the coast (Fig 8). Offshore zooplankton stocks are left largely unaffected by the uniformity in carnivory, as also evident for the Helgoland Roads station (Fig 9) and for area-averages displayed in Fig H in S1 Appendix.

### Effect of viral infections

**Fig 10:**
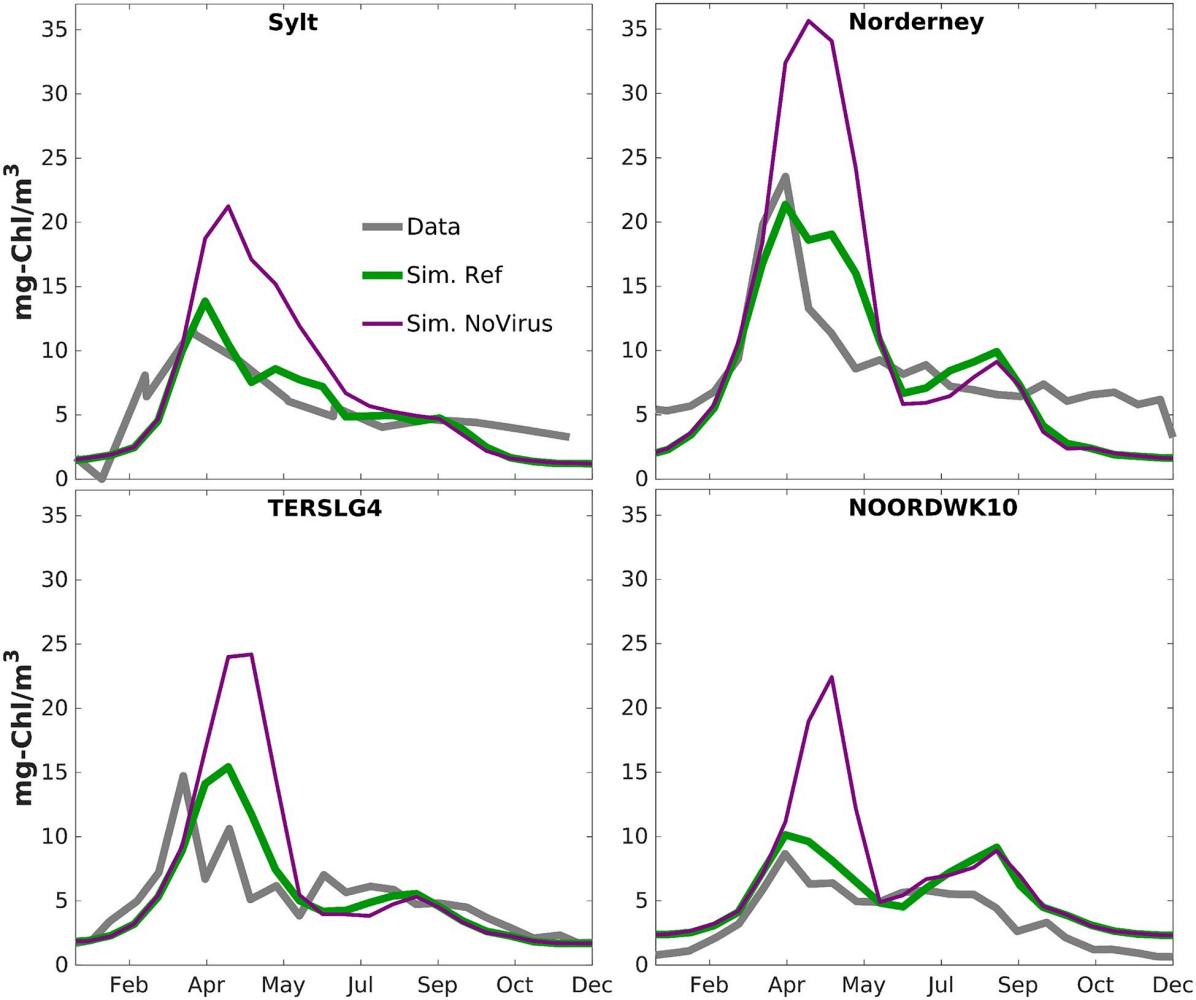
Climatological phytoplankton dynamics. Long-term (2000-2014) averaged chlorophyll concentration at four near-shore stations as calculated from observed time-series (grey), simulated using the standard parametrization (green) or neglecting viral infections (magenta).

In the scenario disregarding virus, growth and decline phases of phytoplankton evolve like in the reference run except for spring when peak CHL concentrations are approximately doubled, as documented in Fig 10 for four stations. Also over the entire SNS, the viral-free spring bloom is much intenser and, most importantly, considerably broader than observed and simulated with the reference configuration (days 115 and 145 in Fig 6). No effects on CHL are discernible in later summer, autumn and early spring. The higher (and unrealistic) spring bloom induces an increase in zooplankton concentration during spring such that measured peak values at Helgoland during early summer are in three of five years moderately better reproduced in that scenario, while in the other two the zooplankton concentration of the reference run is closer to the observations (Fig 9).

## Discussion

### Difference to previous modeling studies

Coastal seas such as the SNS embrace most of the interfaces of regional Earth Systems. Their ecosystems are subject to strong atmospheric, land-borne, and sedimentary drivers, which present a challenge to modeling. Yet, the mostly shallow North Sea is among the best studied regional seas worldwide, also with respect to the diversity of coupled model applications such as MIRO&CO [70], ERGOM-BSHcmod [71], ECOSMO-HAMSOM [19], ECOHAM-HAMSOM [67], ERSEM-BFM-GETM [72], or ERSEM-NEMO [11]. All these models can reproduce major observed trends in ecosystem states such as spring bloom formation or summer nutrient drawdown, but were in general evaluated based on sparse cruise data. Ford et al. used remote sensing data for model validation by comparing ERSEM-BFM-GETM and ERSEM-NEMO to two similar composite CHL images, however only for August 2010 and 2011 [11]. Winter and spring-summer averages were not presented because of unfavorable error statistics. The August composites resemble the respective ESACCI snaphot plotted in Fig 5 (”Aug”, or day 218 of 2010). ERSEM-BFM-GETM qualitatively generated a CHL accumulation towards the continental coast, but with a much lower steepness, lower average concentration and much greater lateral extension, thus often too low values at the coast, in particular in or adjacent to the German Wadden Sea, and a systematic underestimation of offshore surface CHL. ERSEM-NEMO did not even produce any considerable lateral gradient in the North Sea area. The limited skill of ERSEM facing strong coast-shelf variability in CHL is remarkable since ERSEM includes around 7–8 times the number of parameters (∼605) compared to MAECS-OMEXDIA (80), while the horizontal resolution of the GETM simulation by Ford et al. [11] was only by a factor of three coarser than the one presented here. The low skill had motivated implementations of data driven techniques. By assimilating remote sensing data, ERSEM-POLCOMS gained higher accuracy in estimating biogeochemical states of the North Sea [12], what however was still not sufficient to reproduce the slope of cross-shore CHL gradients. Such difficulties to catch remotely sensed CHL distributions, in particular strong cross-shore gradients can be found for coastal-shelf applications around the globe such as Gulf of St. Lawrence [73] or the upwelling system at the U.S. Pacific Northwest coast [9].

### Less is more: Simplicity and model skill

Already a decade ago, the spatial resolution of the MAECS-GETM implementation used here has been reached by the SNS application of BLOOM-Delft3D [74]. BLOOM indeed well reproduced spring blooms at Dutch near-shore stations and steep CHL gradients along the Noordwijk and Terschelling transects in winter and spring, but showed a weaker performance for the summer situation. Special to that model is the lack of a dynamic zooplankton compartment. According to the results presented here, that choice may have positively influenced the overall good model performance as phytoplankton mortality by herbivorous grazing cannot increase towards the coast in BLOOM, as it presumably does in most other shelf ecosystem models where elevated coastal grazing diminishes the CHL gradient. However, lack of grazers may also explain the decreasing skill of BLOOM during summer.

Classical skill measures only offer a limited evaluation criterium for complex ecosystem models. Large uncertainties arise from *ad hoc* decisions on skill metrics, data selection, and data preprocessing or pooling. For example, error statistics differs between an analysis of individual scenes –as for the first time presented in this study—and a global one on the entire remotely sensed data set or on climatological means. Differences in the distribution of skill parameters between different model versions can still be substantial. A similar granularity in skill metrics has already been demonstrated in previous, more extensive calibration studies: An application of ERSEM to the Helgoland monitoring data revealed a distinctively bimodal skill distribution: parameterizations were either “fair” or “off”, without intermediate state [75]. Here, the two carnivory versions performed statistically different, especially during the summer period when zooplankton abundance peaks. Despite an overall large interannual variability, spatial CHL distributions do not vary too much during summer, which leaves not much flexibility for diverse model solutions to match an individual scene, nor to match a seasonally averaged map. This granularity can be considered to withstand the uncertainties in data and metrics such that qualitative interpretations of skill variations between model versions seem possible: variants of MAECS lacking virus or spatially explicit zooplankton mortality were “off”, thus incompatible with major observed system features, which could not be repaired by adjusting individual model coefficients. This result can be taken as indicative –albeit not conclusive– that these or similarly acting factors are relevant also in the real coastal ecosystem.

### Lessons from variable model skill

The credibility of the sensitivity runs crucially depends on the “fair” skill of the presented full version of MAECS-GETM, which already appears from visual inspection and has further been substantiated through classical error statistics based on different pooling strategies. The validation covered a full range of 15 years over the entire SNS, included a quantitative comparison to nearly 400 remote sensing maps and referred to a high density of monitoring stations, however with higher density in the eastern SNS. This monitoring gradient was originally the reason for cutting off the northern North Sea and the Strait of Dover from the model domain, which in turn explains a number of weaknesses at the western edge of the SNS as evident from the comparison to satellite derived CHL images. Given the cut-off, it is surprising that typical features in the western SNS such as the East Anglia plume or variable (non-persistent) CHL accumulation at the UK coast are qualitatively well reproduced by MAECS-GETM, a result that can be attributed to realistic physical boundary conditions and the ability of GETM to keep artifacts from sub-optimal grid lay-outs to a minimum.

The coupled model reproduces steep persistent cross-shore gradients and sporadic long-shore gradients, mesoscale structures, and short-term events in nutrient concentrations and major biological variables. For a more direct assessment of the carnivory hypothesis, usage of additional data related to zooplankton would have been beneficial. Alike for many ecosystem models, the zooplankton state variable in MAECS implicitly resolves both micro– and mesozooplankton grazers what renders the direct comparison to mesozooplankton data more difficult. The slight underestimation of mesozooplankton biomass measured at Helgoland would then indicate an important short-coming since heterotrophic dinoflagellates and ciliates can together reach a concentration of more than 4 mmol-C m^−3^ in early summer [76], what would enlarge the quantitative mismatch. However, the model displays moderately higher zooplankton biomass compared to the CPR data [21]; in addition, an additional microzooplankton model compartment would automatically increase simulated mesozooplankton biomass concentration, since ciliates in part feed on bacteria, but are in turn a preferred food source for copepods.

As an exception to the overall good skill, the model systematically underestimated remotely observed blooms in late autumn and winter. This may indicate a too coarse description of seasonal changes in light climate, or limitations of a single phytoplankton compartment supplemented with a curtailed –since exclusively physiological– representation of community structure changes. Therefore, future model versions should (i) integrate more realistic spatio-temporal variations in turbidity and (ii) test the relevance of other community traits such as cell size as outlined in [77], for better explaining distinct seasonal patterns. This extension would specifically facilitate to (iii) integrate microzooplankton grazing including mixotrophy into the assessment.

### Mortality factors

To date, the standard route of advancing pelagic ecosystem modeling evolves along further investments in model complexity, mostly in terms of additional phytoplankton groups, and increasing spatial resolution of the physical driver model. The example of BLOOM-Delft3D and this study both confirm the necessity of realistic hydrodynamic hindcasts, but at the same time indicate different potential pathways of adding biological realism into modeling. The need for alternative concepts of model building is supported by the fact that an overwhelmingly complex ecological model such as ERSEM struggles to reproduce persistent and major trends in the data. This may be linked to an undervalued role of mortality factors. To a certain extent the relevance of loss functionality also emerges from “simplistic” approaches [74] suggesting that omitting explicit herbivory could yield more realistic predictions than a spatially odd representation. Completeness in the description of mortality factors seems to be as relevant as of of productivity factors, namely light and nutrient fields, which in turn are tightly linked to ocean physics. Equitability of productivity and mortality factors should already be clear from vanishing *net* annual growth rate of most aquatic primary producers as they restart from nearly identical winter concentrations every year. While mortality in biogeochemical models is mostly confined to herbivorous grazing, “end-to-end” models provide a richer discription of herbivores as these are subject to predation by carnivores, usually small fish. However, the major goal of these “end-to-end” modeling studies is to obtain prey fields for simulating fish population dynamics [19, 20], and not so much to better understand spatio-temporal variability in phytoplankton.

A large part of temporal variability in phytoplankton biomass consolidates at the seasonal scale. While the spring bloom in general defines the major signal in temperate seas, near-coast areas frequently display a summer bloom, or two of them [78, 79, 80]. As a consequence, a clear water phase with sometimes extremely low CHL in late spring/early summer defines a second major seasonal event in the phytoplankton distribution, similar to many lakes. The depression in CHL after the spring bloom has frequently been attributed to zooplankton grazing. This hypothesis has its roots in the seminal work of Riley [81] and was recently reframed by Behrenfeld [82], who still underlined the relevance of top-down control. However, grazing as ultimate cause of the phytoplankton stock removal is incompatible with the observation that zooplankton in late May/early June is still an order of magnitude below its concentration peak around July. Herbivorous grazing has a rather limited impact on spring bloom termination also in other coastal systems such as the Chesapeake Bay [83].

### Viral bloom control

Mortality factors comprise both ecological and physiological aspects. The latter are here featured through a novel virus module. The impact of viral dynamics is found to be limited to the post-bloom period, at least for most simulation years, where viruses effectively reduce peak phytoplankton concentrations by 30–50%. Virus-free simulations thus significantly overrate late spring and early summer CHL. This may not appear a strong result since models could try to repair for neglected pathogens by increasing other mass loss factors such as aggregation/sedimentation, respiration/exudation, or grazing. However, other loss factors are more homogeneously distributed in time compared to the viral bloom in late spring so that their increase raises model-data deviations during the early spring bloom and during late summer and autumn. A slightly better agreement between the zooplankton data at Helgoland and the respective results of MAECS in the virus-free simulations thus indicates weaknesses in the grazing parametrization and not a better description for phytoplankton losses as the larger zooplankton stock originates from overrated phytoplankton concentrations. Also, qualitative comparison with the CPR zooplankton data indicates a better skill of the reference run compared to other scenarios. It is still conceivable that an explicit account of fast growing microzooplankton would induce similar effects like the virus implementation introduced here. This implementation contributed to the narrowing of the near-shore band of high CHL by turning down intermediate phytoplankton levels in the transitional zone, which together with the realistic dampening of the spring bloom renders a strong viral control on phytoplankton communities at least compatible with the data. Strong viral control of the decline is compliant with the event-scale observation of a coccolithophore bloom in the North Atlantic [84]. At Helgoland, virioplankton density in bulk seawater has been reported to vary intermittently and to peak during late spring and early summer [85], which is qualitatively in line with the model outcome for host–attached virions. Yet, without more extensive quantitative field data it will remain difficult to thoroughly test virus-plankton models.

### Modeling concepts for host-phage dynamics

Viruses are frequently observed in phytoplankton cells and are therefore potential major loss factor to be considered when studying ecosystem dynamics [24, 25, 86, 26]. Simulated mortality rates of about 0.25d^−1^ over 2–3 months as quantified here constitute a major, partially dominant stress factor for phytoplankton populations, refining previous estimates of viral induced mortality that point to a similar quantitative impact as (microzooplankton) grazing [60, 61]. A challenge of incorporating viral lysis into ecosystem modeling derives from its short term dynamics [87], but also from the complicated pathways with various life-stage and species–specific dependencies. More complete accounts of viral infections were therefore tempted by conceptual models of virus–host interactions that distinguish between different infection states or feature species– and group specificity [88, 87, 27]. In comparison, this work proposed an approach of reduced complexity that nonetheless generated the often intermittent type of infection dynamics and, for the first time, resolved adaptive immune responses and dependencies on physiological factors such as on intracellular nutrient stoichiometry. Especially the novel, mechanistic and slim implementation of anti–pathogen defense is sufficiently generic such that it should describe a larger spectrum of damaging symbionts, diseases, or parasites, both with respect to similar deleterious effects and defense costs. The approach gains importance for the overarching goal of assessing climate change effects in marine ecosystems because non-negligible pathogenic control would modify the sensitivity to temperature. For example, warming has been suggested to promote growth of fungal parasites and thus the termination of the phytoplankton spring bloom [89].

### Physical imprints on lateral distribution of nutrients and turbidity

Conceptual model studies indicate that asymmetric tidal transport by density-driven estuarine circulation and tidal pumping is the main driver of coastal gradients in nutrient levels and SPM [16, 39]. The accumulation of organic and inorganic material in near-coast waters is presumably further enhanced by raised particle settling rates in the coastal transition zone, as recently found for the eastern SNS based on Scanfish data [44]. Asymmetric tidal transport and detritus accumulation is inherent to our realistic GETM simulations, which –except for summer– induces a steep increase in nutrient concentration independently from the distance to a major estuary. This nutrient increase potentially stimulates phytoplankton growth, but is counteracted by the parallel increase in SPM and thus light attenuation.

It would of course be more consistent to simulate SPM dynamics alongside with ecosystem dynamics as attempted within ERSEM-BFM-GETM and ERSEM-NEMO, but their skill for SPM turns out even worse than for CHL such that a simple parametrized representation like used here still appears a reasonable choice. The spatio-temporal pattern of turbidity is commonly known to be key for coastal primary production [6], as also demonstrated for the transitional waters of the SNS [90]. However, a predecessor version of MAECS assumed a cross-shore doubling of total background attenuation (including bio–shading) from offshore areas to near-shore [33], in contrast to the accumulation factor of more than ten estimated by remote sensing. The too flat gradient originates from reliance on turbidity forcing data [67] predicting merely a 40% increase. Owing to the unrealistic account of the coastal light climate, the predecessor version [33] was able to generate a coastal CHL gradient –albeit not as steep as observed– without assuming coastal accumulation of carnivory. In contrast, the attenuation distribution used here not only derives from *in situ* Scanfish data [44] but is in addition compliant with the ESACCI data displayed in [33] as well as SPM modeling results [30, 32].

### Independence of biology from physics

Strong near-coast physical drivers such as asymmetric tidal transport create significant cross-shore gradients but with compensating effects on primary productivity. Asymmetric transport will furthermore only be relevant in relatively shallow shelf-coast systems, whereas productivity gradients in areas with steeper continental slopes are in general dominated by nutrient input through upwelling so that the findings of this study on biological drivers may not fully apply there. Yet, also in those systems, pathogens and lateral heterogeneity in carnivorous grazing can be thought to compete with physical drivers. For example, the slope of the observed CHL gradient at the Pacific Northwest coast exceeds the predicted one based on an otherwise realistic hydrodynamics [9], and lateral variability in upwelling frequency only weakly correlates with the one in CHL in the California Bight [14], supporting the picture of a complex mixture of physical and non-physical processes [91]. The lateral distribution of phytoplankton will therefore only partly reflect physical drivers, which has also been shown by spectral analysis of transect data for the North Atlantic [92].

### Coastal gradient in herbivorous grazing

Coastal amplification of turbidity not only hampers primary production, but also constrains secondary producers in their search for food: visible ranges of few decimeters provide protection against predators such as fish or birds. Feeding limitation of fish by turbidity as been reported in, e.g. [49, 51], which will provide fish juveniles more shelter in turbid waters near to the coast [52, 53]. As a consequence, specific zooplankton mortality will increase with rising turbidity, an assumption also inherent to the pattern proposed by Maar et al. [21].

Furthermore, a largely overlooked ecological mortality source originates from bivalves and other suspension feeders. These benthic animals concentrate at the coast [93], and were so far incorporated into ecological models such as ERSEM as strict herbivores. However, mussels ingest high numbers of copepodites and –to a variable degree– adult copepods [55, 56, 57]. [58] therefore elaborated a possible contribution of benthic filter feeders to near-coast carnivory. Here, I provided indirect evidence for enhanced relevance of benthic predation not only for regulating phytoplankton stocks but more so the herbivores. Estimated specific mortality rates due to suspension feeding mostly falls below 0.1d^−1^, which is small in relation to typical autotrophic growth rates but large for relatively slow growing herbivores. Using a set-up nearly identical to the one presented here, a simple filtration module coupled to MAECS calculated a reduction in surface CHL of about 20% for water column depth below 15m [94]. This motivates further investigations where suspension feeding also removes mesozooplankton. Future model studies not only need to explicitly resolve the spatial distribution of both small fish and benthic filter feeders, but should also incorporate a wider range of trophic interactions. As already stated above, mixotrophs and microzooplankton may occasionally become relevant in the German Bight [76], which would give rise to an alternative food chain from pico-phytoplankton, small heterotrophs to mesozooplankton, where increased near-shore predation on copepods may have a negative effect on small phytoplankton species. However, measurements at Helgoland suggest that a significant portion of phytoplankton biomass falls in the feeding size range of copepods between 10 and 40μm cell diameter, at least during spring [95] or during most of the growing season (Apr-Sep) [96]. Cascading effects of carnivory on mesozooplankton still remain open in cases where coastal blooms are dominated by either pico-phytoplankton or huge diatoms.

The presented sensitivity of non-uniform carnivory for coast-shelf variability may yet point to significant system effects, also because it extends to a massive imprint on biogeochemistry given the alterations in simulated summer nutrient concentrations at many coastal sites by roughly one order of magnitude. In contrast to most previous ecosystem models applied to the North Sea, the model version resolving variable carnivory also features strong summer blooms, most prominently in the transition zone of semi-stratified waters. The carnivory increase constitutes not only the most probable but also most simple process to prevent strong accumulation of pelagic herbivores at the coast and turned out to be the most effective mechanism to generate and maintain CHL gradients. The concomitant decoupling between primary production and herbivory agrees with studies in other systems. At the mouth of the eutrophic Pearl river estuary, copepods exerted a moderate predation pressure on highly abundant phytoplankton stocks [97], which was hypothesized to reflect poor food quality and increased predation in the estuarine waters [98]. In the Bay of Biscay, the zooplankton distribution pattern shows no correlation to lateral gradients in primary production [99]. For the eastern SNS, zooplankton biomass in the simulation presented here smoothly increases from offshore (in average >1-2 mmol-C m^−3^) to near-shore (<4 mmol-C m^−3^), which is compatible with spatially coarse empirical reconstructions for single key species [100, 101, 53].

### Conclusion

Marine and coastal ecosystem research is since long puzzled by strong and irregular variability in biological observables [102], which is here made evident by a large number of independent observations. Matching such multi-scale variability demonstrates an unprecedented skill of a coupled model. This skill not only reflects an important advancement in our search for key mechanisms shaping coastal ecosystems, but also formed the basis for numerical experiments that highlight the role of processes barely represented in classical ecosystem models. Exacerbated bloom peaks during spring are prevented by short term host-pathogen dynamics that comprises viral infection and antiviral defense. Strong biomass reduction in a coastal transition zone due to pathogens in particular strengthens the sharp and ubiquitous cross-shore CHL gradient. Most importantly, this gradient only emerges when assuming an increase in carnivory towards the coast. The latter process can be well justified based on literature accounts of both the behavior of juvenile fish and settlement patterns of benthic filter feeders. However, fish and benthic organisms show other mobility behaviors than passively drifting plankton, which further challenges Eulerian ocean models. In total, this joint model–data study unravels a substantial independence of biological variability from physical factors.

## Supporting information

S1 Appendix Text A, Benthic biogeochemistry. Text B, Viral dynamics: infection, replication, and mortality. Fig A, Dependencies of model parameters on water column depth. Fig B, Remote sensing chlorophyll-a (CHL) plotted against *in situ* data from Dutch and German marine monitoring stations in the southern North Sea. Fig C, Climatological average (2000-2014) of simulated nutrient concentrations. Fig D, Long-term dynamics of DIN at 7 stations in the southern North Sea. Fig E, Same as Fig D for DIP. Fig F, Climatological averages (2000-2014) of the seasonal distribution in surface CHL. Fig G, Distribution of the correlation between simulated and remotely sensed CHL maps of the SNS. Fig H, Climatological seasonality in simulated zooplankton concentration. Fig I, Climatological summer distribution in simulated surface CHL. Table A, Model parameters of the new MAECS and OMEXDIA variants. Table B, Zooplankton weight estimates.
(PDF)
