# Supplementary material for: Physics or biology? Persistent chlorophyll accumulation in a shallow coastal sea explained by pathogens and carnivorous grazing
Source: PLoS One. 2019 Feb 22;14(2):e0212143. doi: 10.1371/journal.pone.0212143 (PMC6386242; doi:10.1371/journal.pone.0212143)
Supplement: S1 Appendix — Text A, Benthic biogeochemistry. Text B, Viral dynamics: infection, replication, and mortality. Fig A, Dependencies of model parameters on water column depth. Fig B, Remote sensing chlorophyll-a (CHL) plotted against in situ data from Dutch and German marine monitoring stations in the southern North Sea. Fig C, Climatological average (2000-2014) of simulated nutrient concentrations. Fig D, Long-term dynamics of DIN at 7 stations in the southern North Sea. Fig E, Same as Fig D for DIP. Fig F, Climatological averages (2000-2014) of the seasonal distribution in surface CHL. Fig G, Distribution of the correlation between simulated and remotely sensed CHL maps of the SNS. Fig H, Climatological seasonality in simulated zooplankton concentration. Fig I, Climatological summer distribution in simulated surface CHL. Table A, Model parameters of the new MAECS and OMEXDIA variants. Table B, Zooplankton weight estimates. (PDF) [file pone.0212143.s001.pdf]

Supporting Information for "Physics or biology? Persistent chlorophyll accumulation in a shallow coastal sea explained by pathogens and carnivorous grazing"

Kai W. Wirtz[a]

[a]*Institute of Coastal Research, Helmholtz-Zentrum Geesthacht, Geesthacht, Germany.*

Contents of S1 Appendix

1. Text A to B
2. Figures A to I
3. Tables A to B

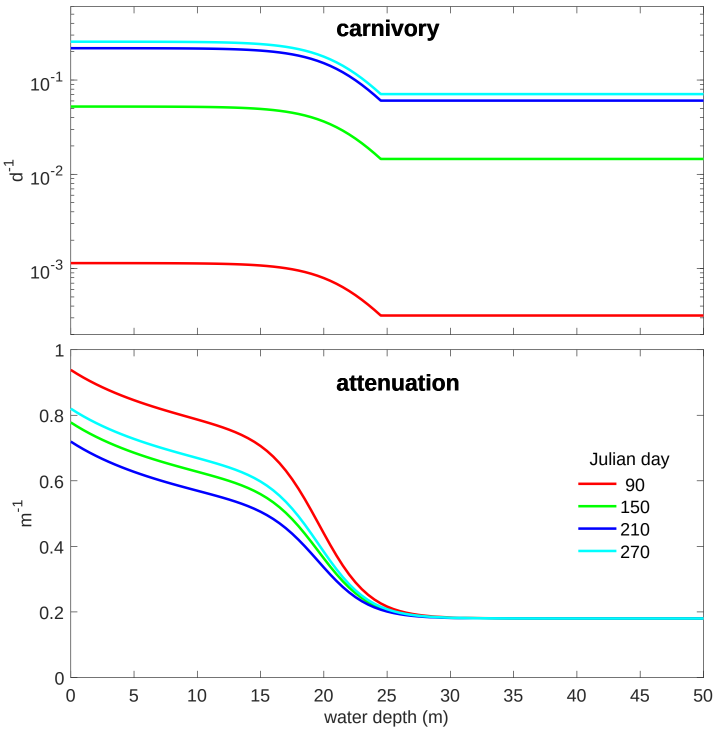

Fig A: **Dependencies of model parameters on water column depth.** Water depth can be taken as a proxy for the distance to the coast. The curves also vary with season, here from early spring (Julian day 90) to early autumn (day 270). Top: Specific zooplankton mortality rate m_{Z} at constant salinity (S=31 in Eq.(4)). Bottom: Light attenuation without the contribution of organic particles, thus emulating the adsorption by lithogenic particles and CDOM.

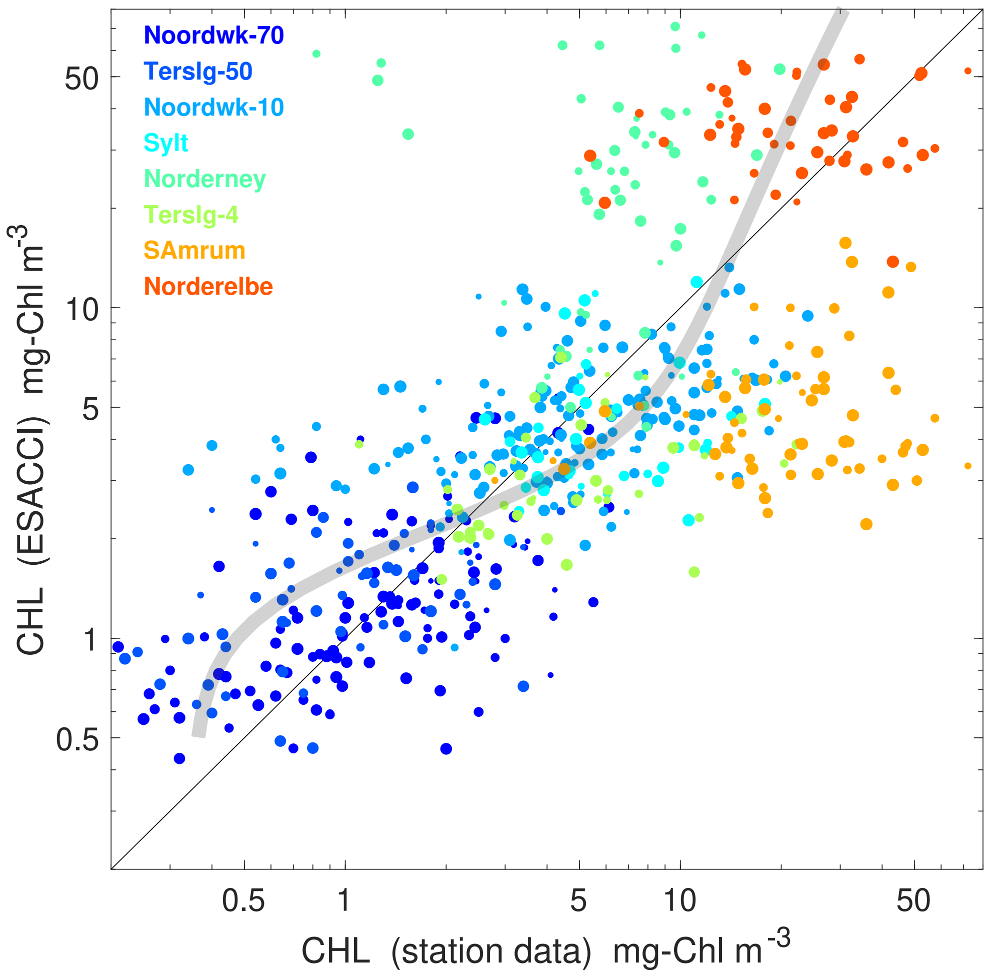

Fig B: **Remote sensing chlorophyll-a (CHL) plotted against *in situ* data from Dutch and German marine monitoring stations in the southern North Sea.** The circle diameter indicates the time difference between the two measurements from below 1 day (larger circles) to 3 days (smallest circles). A non-linear transformation of the ESACCI CHL seeks to better fit the *in situ* data while allocating equal weight to each station, thus trying to minimize the error to the cluster center of each station data set (broad grey line): CHL=$0.35 \cdot 10^{1.2c^{3.1}/(8+c^3)}$ with c denoting the ESACCI CHL in mg-Chl m^{-3}.

A. Benthic biogeochemistry

A variant of OMEXDIA (Soetaert et al., 1996) brought into the FABM format simulates diagenetic processes in the sea floor. The original version of the diagenesis model calculates a simplified carbon and nitrogen cycle, primarily consisting of respiration of benthic particulate organic matter (bPOC), nitrification and denitrification as visualized in Fig 2. In addition, the P cycle is represented by the turnover of a dissolved species, bPO4, and a particulate pool bPP comprising both inorganic and organic forms. Adsorption of bPO4 (transformation into the particulate pool bPP) ceases when a critical capacity of electron donators (C_{PAds}, see Table A in S1) is reached, which in turn is given by the difference between oxygen demand units, bODU (a pool of reduced substances, e.g., iron and hydrogen sulfide), and dissolved oxygen concentration, bO2 (benthic oxygen concentration):

$$\frac{\mathrm{d}}{\mathrm{d}t}\mathrm{bPO4} = f_T\, r_{\mathrm{P}}\, \mathrm{bPP} - r_{\mathrm{PAds}}\, \mathrm{bPO4} \cdot \sigma_{s_{\mathrm{PAds}}}(C_{\mathrm{PAds}} + \mathrm{bODU} - \mathrm{bO2}) \qquad (1)$$

with the sigmoid response function $\sigma_s(x)$ defined in Eq.(3). The time derivative of bPP is equal to Eq.(1) with negative sign.

Numerical instabilities in the original variant of OMEXDIA are prevented through an alternative management of the oxygen dynamics. Immediate oxygen demand in OMEXDIA reflects the concentration of NH3, high quality bPOC, and bODU. Oxygen consumption therefore contains aerobic respiration of high and low quality bPOC, nitrification, as well as oxidation of reduced substances, and depends on a saturation function of bO2 with process specific half-saturation constants. These constants are in the new model version raised by a small relaxation parameter times the concentration of the competing oxygen pathways (e.g., bPOC and bODU for NH3 oxidation). This scheme downscales consumption rates under high competition of electron donators at low levels of bO2, which in the numerical integration scheme prevents the tendency towards negative bO2 under such conditions.

B. Viral dynamics: infection, replication, and mortality

The dynamics in intracellular viral density v is here described by three aggregate processes, (1) infection–replication, (2) virus removal by (selective) host reactions, and (3) virus mortality.

(1) Infection comprises multiple stages which entail processes outside and inside the host organisms. Viral adsorption rate at the cell membrane r_{ads} is proportional to a specific rate r^*_{ads} (likely influenced by water turbulence), and to the bulk virus concentration in the water, which is in the model proportional to the internal density v times the phytoplankton concentration PhyC, thus $v \cdot \mathrm{PhyC}$, and to the ratio of cross-sections describing the chance of hitting an autotrophic host. The cross-section ratio relates the probability for the virus to collide with a phytoplankton cell (increasing with PhyC) to the collision probability for other particles such as bacteria, aggregates, or lithogenic material.

$$r_{\mathrm{ads}} = r^*_{\mathrm{ads}} \cdot v \, \mathrm{PhyC} \cdot \frac{\mathrm{PhyC}}{\mathrm{POC}} \tag{2}$$

Hence, high concentrations of detritus protect phytoplankton cells, in line with the observed reduction in infection rate with increasing abundance of bacteria and colloids (Murray, 1995). However, the real effect might be stronger as the lithogenic contribution is neglected in the POC calculation.

After infection, intracellular viral replication using either RNA or DNA of the host cell depends on temperature (Brussaard, 2004) and the carrying virus capacity of the (decaying) cell, which analogue to Eq.(6) is a smooth step function $\sigma_s(v_{\max} - v)$ with capacity $v_{\max}$. The same non-linear form is here assumed to apply to the dependency on the physiological state of the host cell as expressed by its C:N:P stoichiometry (Hadas et al., 1997; Clasen and Elser, 2007; Birch et al., 2012) as proxy for protein and RNA/DNA turnover. The C:N:P stoichiometry is in MAECS given by relative N- and P–quotas (q_{N} and q_{P}, see Wirtz and Kerimoglu (2016)). Replication then should cease for low N- and P–quotas and reach a maximum for sufficiently

high quotas. Together, we have

$$r_{\text{rep}} = n_v^* f_T \, \sigma_s(q_{\text{N}} - 1) \, \sigma_s(q_{\text{P}} - 1) \, \sigma_s(v_{\max} - v) \tag{3}$$

with maximal burst size n_v^*. The latter can be combined with the specific adsorption rate r_{ads}^* into the viral maturation rate r_v^*.

(2) Viral density within a host population declines due to endogenous mortality. The corresponding rate r_{mort} increases with temperature but ceases at very low density ($v < v_{\text{low}}$), with which the model imposes a persistent background or latent virus concentration in the environment,

$$r_{\text{mort}} = r_{\text{mort}}^* \, f_T \, \frac{v}{v + v_{\text{low}}} \cdot v \tag{4}$$

The specific rate r_{mort}^* can be estimated from viral residence times in the surface ocean reported to be on the order of 1 day (Suttle and Chen, 1992). (3) Viral density in addition decreases because of antiviral defense such as preferential decline of more infected hosts. I here introduce a mathematical expression for preferential host mortality, which should also describe the more general case of host defense. Infected phytoplankton often displays apoptosis, i.e. programmed cell death (Bidle and Falkowski, 2004). As a consequence of the survival of healthy or less infected individuals or groups/species, average pathogen density v decreases. Preferential mortality of infected individuals and concomitant "cleaning" of the population can be understood as competitive process. Within the general formalism of competitive trait changes introduced by Wirtz and Eckhardt (1996) , the resulting decline rate, r_{defense}, is proportional to the diversity of infection degrees within the host population (δ_v) and the marginal growth change of hosts due to viral disease. This marginal growth change is given by the (negative) derivative of the phytoplankton mortality $m_{\text{P,vir}}$ with respect to viral density v, thus -$\partial m_{\text{P,vir}}/\partial v$, or with Eq.(6), $m_{\text{P,vir}}^* \, s \, \sigma_s^2 \; \mathrm{e}^{s \cdot (1-v)} = m_{\text{P,vir}} \, s \, \sigma_s \; \mathrm{e}^{s \cdot (1-v)}$.

Diversity of infection degrees has been derived for (trait) variables strictly

bounded between zero and $v_{\max}$ to be proportional to $v \cdot (v_{\max} - v)$ (Wirtz and Eckhardt, 1996). According to our definition of v, such a biomechanical upper bound will be around $v_{\max}$=2. In addition, the diversity in host infection degrees depends on the structure of the host community. A community dominated by few species with relatively homogeneous infection level, displays a small diversity, whereas coexistence of a broad variety of species and thus pathogenic affinities and histories will increase infection diversity. This specificity is here estimated refering to studies on biomass–diversity relationships. For example, the data of phytoplankton species diversity presented by Interlandi and Kilham (2001) can be well fitted by an inverse function of total phytoplankton biomass (here $\mathrm{e}^{-\mathrm{PhyC}/\mathrm{C}_{\mathrm{low}}}$), such that both host and disease diversity will be low at high total biomass, which will render countermeasures such as apoptosis more effective. Finally, I assume any defense mechanism to cease at very low (but non-zero) viral density v_{low} like for virus mortality above. Together, virus removal by preferential hosts mortality, is

$$r_{\mathrm{defense}} = \delta_v \frac{\mathrm{d}m_{\mathrm{P,vir}}}{\mathrm{d}v} = v \cdot (v_{\max} - v) \mathrm{e}^{-\mathrm{PhyC}/\mathrm{C}_{\mathrm{low}}} \cdot \frac{v}{v + v_{\mathrm{low}}} \cdot m_{\mathrm{P,vir}}\, s\, \sigma_s\, \mathrm{e}^{s} \cdot (1 - v) \tag{5}$$

Notably, Eq.(5) can also be thought to describe the expression of defense machinery or occurrence of virophages; anti-viral defense will increase with the marginal impact of the disease. This response will be most pronounced at lethal infection level v=$v_{\max}/2$=1 where the defense rate r_{defense} is around $2m_{\mathrm{P,vir}}$ at high host biomass.

Table A: Model parameters of the new MAECS and OMEXDIA variants.

Symbol	Description	Value	Unit	Source
a'_{SPM}	specific attenuation coefficient	1	m^{-1}	
α	contribution of SPM to a_{SPM}	0.18		(Maerz et al., 2016)
ϵ^*	critical bottom turbulent energy dissipation rate for resuspension	$1.3\ 10^{-3}$	$\mathrm{m}^2\mathrm{s}^{-2}$	Yuan et al. (2008), (Nasermoaddeli et al., 2018)
H^*	depth threshold for resuspension	20	m	(Maerz et al., 2016; Nasermoaddeli et al., 2018)
m'_{Z}	specific zooplankton mortality	0.025	d^{-1}	Maar et al. (2014)
γ	quadratic zooplankton mortality	0.1	$\mathrm{m}^3(\text{mol-C d})^{-1}$	
β	amplitude of lateral variations in zooplankton mortality	18		
v_{low}	minimum (latent) viral density	0.0005		
v_{max}	maximal virus capacity	2		
r_v^*	specific viral maturation rate	0.3	d^{-1}	
r_{mort}^*	mortality of viruses	0.15	d^{-1}	
$\mathrm{C}_{\mathrm{low}}$	phytoplankton biomass to scale infection diversity	2	$\text{mol-C}\,\mathrm{m}^{-3}$	
s	steepness of viral dose-response curve	4		
r_{P}	specific degradation rate of labile POP	0.16	d^{-1}	
C_{PAds}	critical electron acceptor capacity for P-adsorption	200	$\text{mol-}O_2\,\mathrm{m}^{-3}$	
s_{PAds}	steepness of P-adsorption relationship	0.025		
r_{PAds}^*	specific P-adsorption rate	4	d^{-1}	

Table B: Zooplankton weight estimates for converting from counts to biomass data using HTTP://COPEPODES.OBS-BANYULS.FR and Blaxter et al. (1998).

Species/group	µmol-C/Ind
Acartia spp.	0.3
Temora longicornis	0.9
Para- and Pseudocalanus	0.6
Oithona	0.3
Centropages hamatus	0.55
Centropages spp.	0.55
Calanus spp.	2.5
Euterpina	0.06
Tisbe	0.06
Metridia lucens	1.99

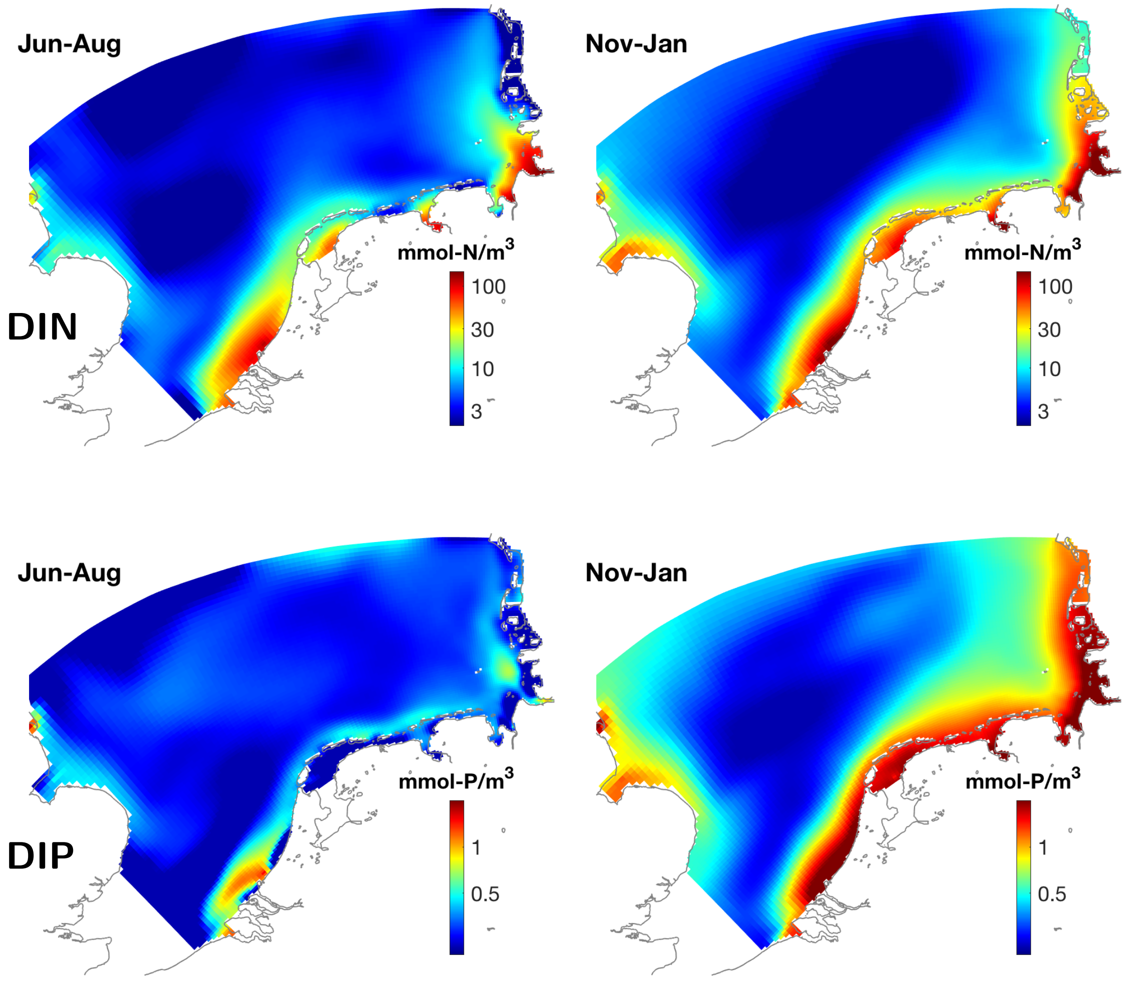

Fig C: **Climatological average (2000-2014) of simulated nutrient concentrations.** Summer (left column) and winter (right) distribution in dissolved inorganic nitrogen (DIN, upper row), and dissolved inorganic phosphorus (DIP, bottom) simulated by MAECS for the surface layer of the southern North Sea.

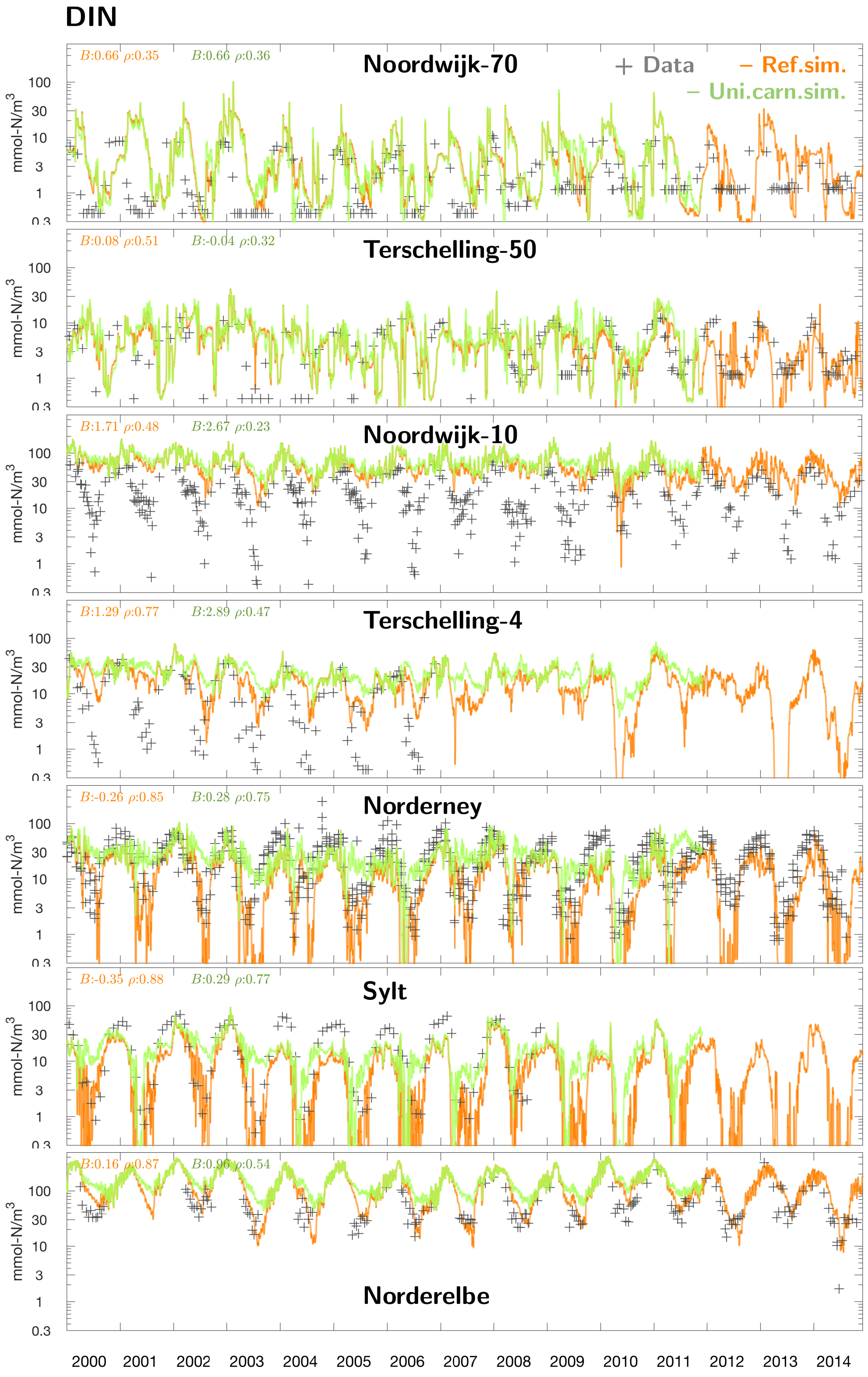

Fig D: **Long-term dynamics of DIN at 7 stations in the southern North Sea.** The stations are ordered according to the distance to the coast with the most offshore station at the top. Compiled measurements (gray crosses) are compared to the reference run (orange line) and the run with spatially uniform carnivory (green line). This scenario is only plotted until end of 2011 for better visibility. Normalized bias B and correlation coefficient ρ of the model–data comparison are added in the color of the respective scenario.

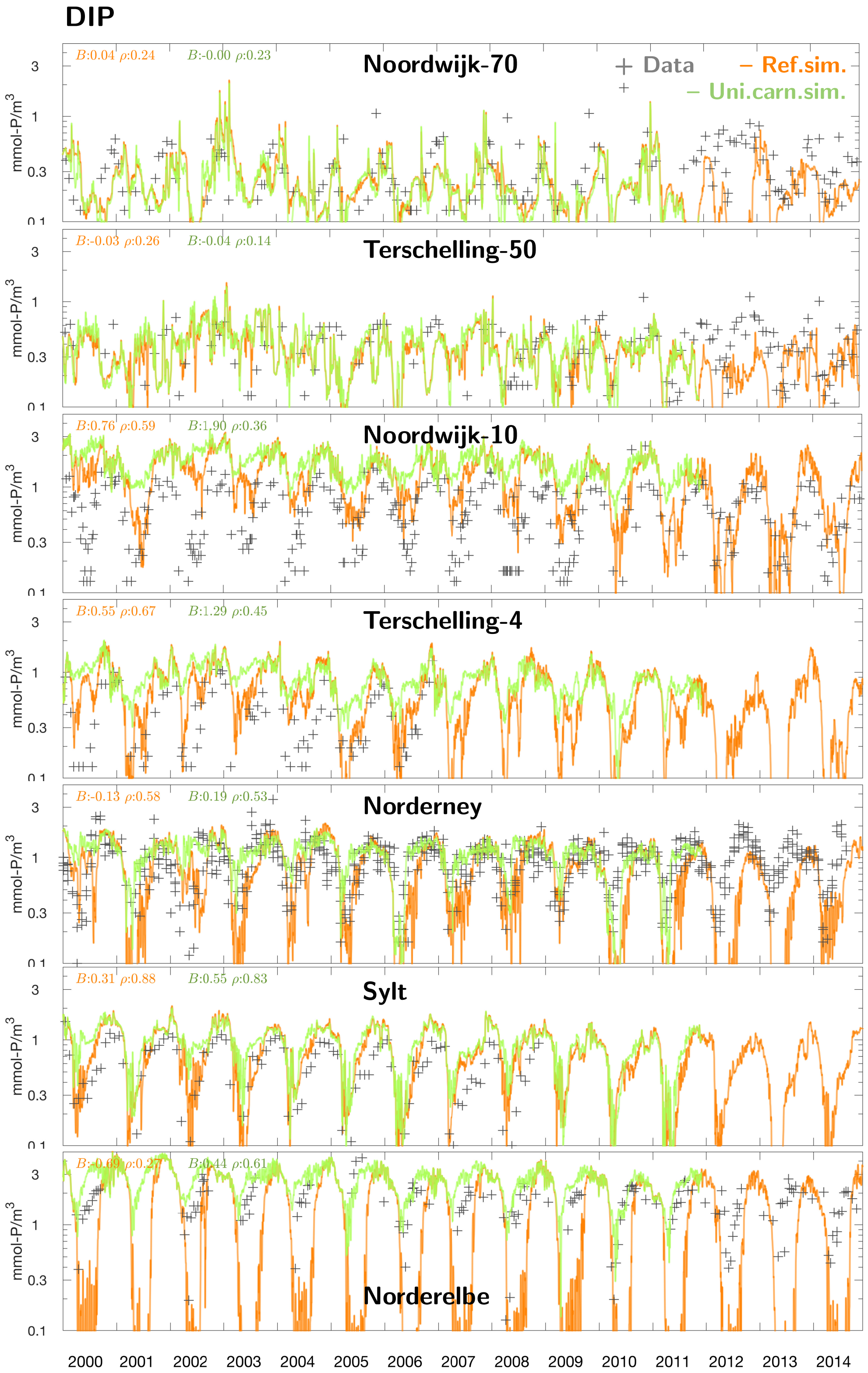

Fig E: same as Fig D in S1 for DIP.

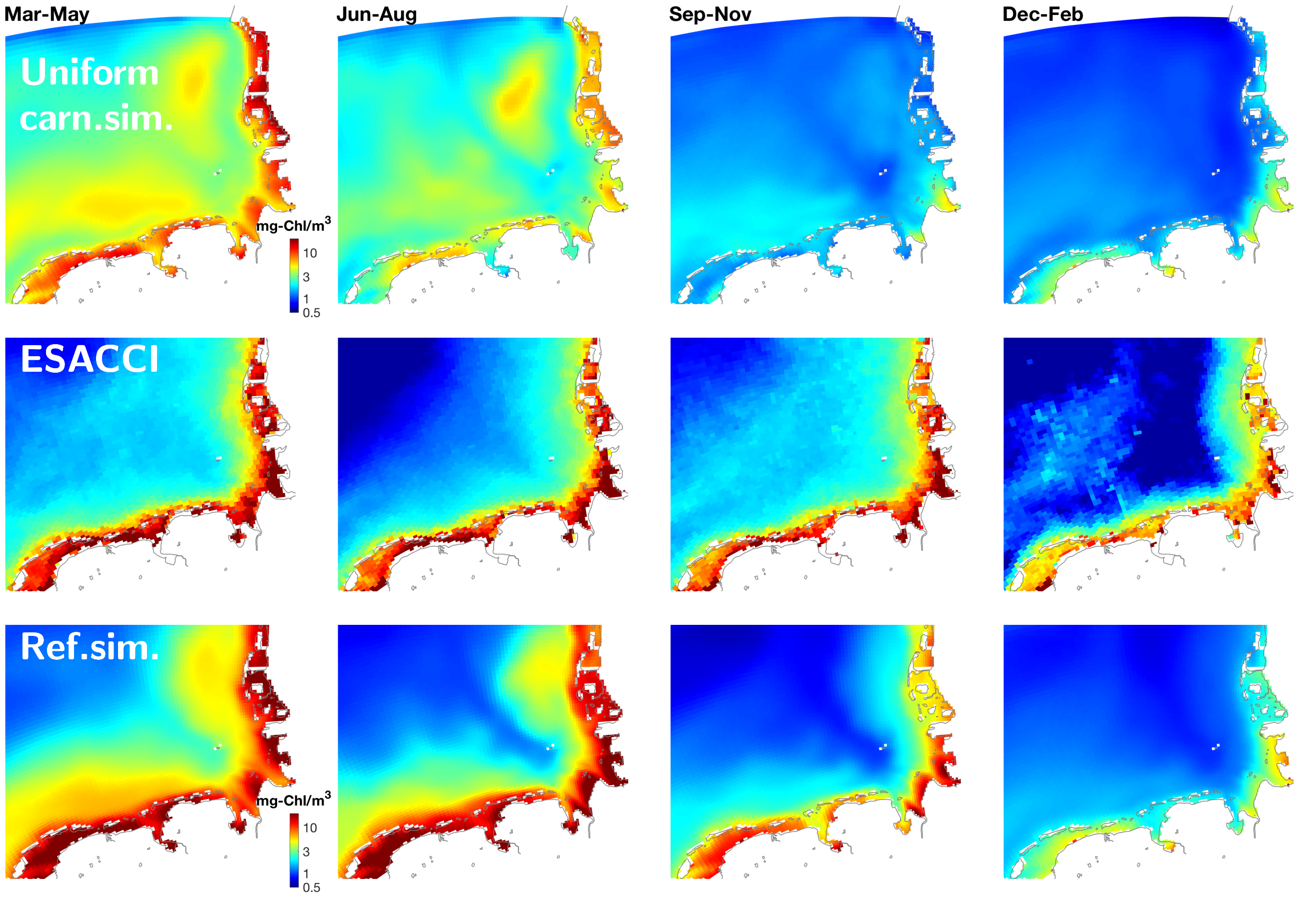

Fig F: **Climatological averages (2000-2014) of the seasonal distribution in surface CHL.** The reference simulation (bottom row) is compared to remote sensing data from ESACCI (middle) and the scenario of uniform carnivory (top).

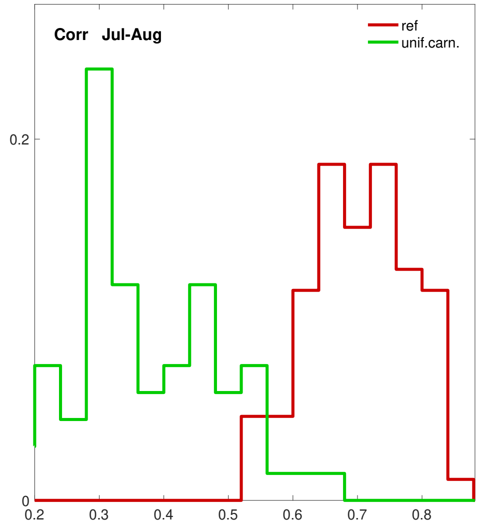

Fig G: **Distribution of the correlation between simulated and remotely sensed CHL maps of the SNS.** Individual correlation coefficients were calculated using ESACCI scenes within two summer month (Jul-Aug, 2000-2014) and CHL maps of the reference run (red) and of the run with spatially uniform carnivory (green).

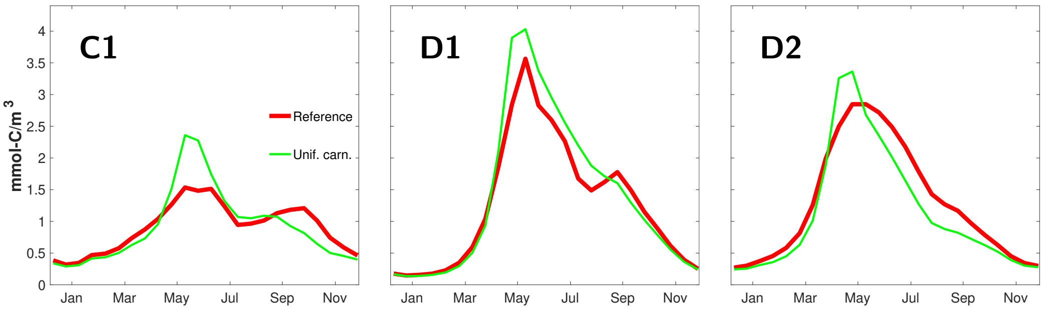

Fig H: **Climatological seasonality in simulated zooplankton concentration.** Averages were calculated for the period 2001-2004 over three CPR standard areas (C1, D1, and D2, see Maar et al. (2014)) based on the reference run (red) and the "uniform carnivory" scenario (green).

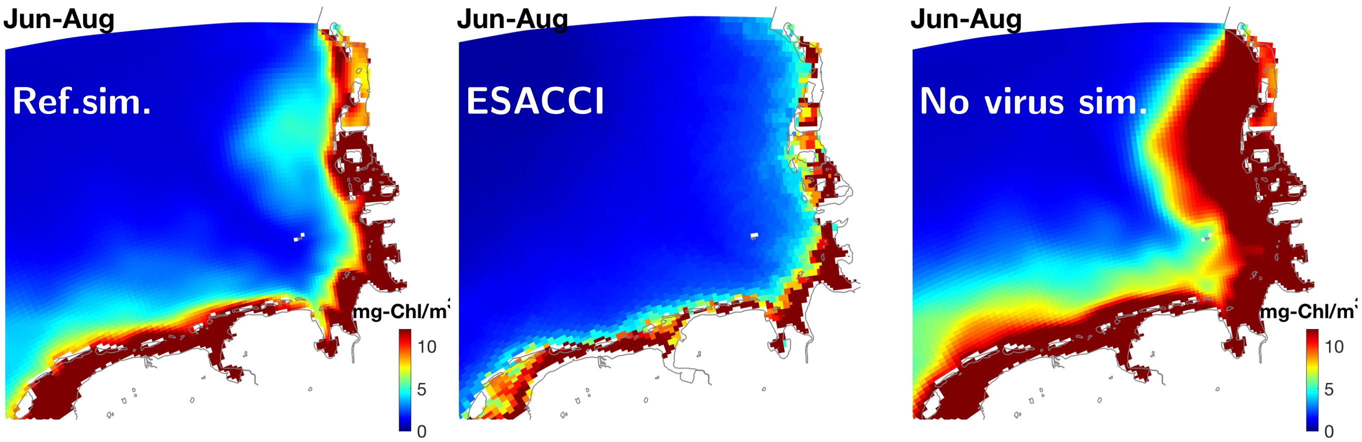

Fig I: **Climatological summer distribution in simulated surface CHL.** The reference simulation (left) is compared to remote sensing data from ESACCI (center) and the scenario of lacking viral infections (right). In contrast to other maps, CHL is plotted using a linear (non-logarithmic) scale.

Bidle, K. D., Falkowski, P. G., 2004. Cell death in planktonic, photosynthetic microorganisms. Nature Reviews Microbiology 2 (8), 643–655.

Birch, E. W., Ruggero, N. A., Covert, M. W., 2012. Determining host metabolic limitations on viral replication via integrated modeling and experimental perturbation. PLOS One 8 (10), e1002746.

Blaxter, J. H., Douglas, B., Tyler, P. A., Mauchline, J., 1998. The biology of calanoid copepods. Vol. 33. Academic Press.

Brussaard, C. P., 2004. Viral control of phytoplankton populations–a review. J. Eukaryotic Microbiol. 51 (2), 125–138.

Clasen, J. L., Elser, J. J., 2007. The effect of host chlorella nc64a carbon: phosphorus ratio on the production of *Paramecium bursaria Chlorella* virus-1. Freshw. Biol. 52 (1), 112–122.

Hadas, H., Einav, M., Fishov, I., Zaritsky, A., 1997. Bacteriophage T4 development depends on the physiology of its host *Escherichia coli*. Microbiology 143 (1), 179–185.

Interlandi, S. J., Kilham, S. S., 2001. Limiting resources and the regulation of diversity in phytoplankton communities. Ecology 82 (5), 1270–1282.

Maar, M., Rindorf, A., Møller, E. F., Christensen, A., Madsen, K. S., van Deurs, M., 2014. Zooplankton mortality in 3D ecosystem modelling considering variable spatial–temporal fish consumptions in the North Sea. Progr. Oceanogr. 124, 78–91.

Maerz, J., Hofmeister, R., van der Lee, E. M., Gräwe, U., Riethmüller, R., Wirtz, K. W., 2016. Maximized sinking velocities of suspended particulate matter in a coastal transition zone. Biogeosciences 13 (17), 4863–4876.

Murray, A. G., 1995. Phytoplankton exudation: exploitation of the microbial loop as a defence against algal viruses. J. Plankton Res. 17 (5), 1079–1094.

Nasermoaddeli, M. H., Lemmen, C., Hofmeister, R., Stigge, G., Klingbeil, K., Kösters, F., Wirtz, K. W., 2018. Large-scale effect of macrofauna on the suspended sediment concentration in a shallow shelf sea (southern North Sea). Est. Coast. Shelf Sci. 211, 62 – 76.

Soetaert, K., Herman, P. M., Middelburg, J. J., 1996. A model of early diagenetic processes from the shelf to abyssal depths. Geochim. Cosmochim. Acta 60 (6), 1019–1040.

Suttle, C. A., Chen, F., 1992. Mechanisms and rates of decay of marine viruses in seawater. Applied and Environmental Microbiology 58 (11), 3721–3729.

Wirtz, K., Kerimoglu, O., 2016. Autotrophic stoichiometry emerging from optimality and variable co-limitation. Frontiers Ecol. Evol. 4, doi:10.3389/fevo.2016.00131.

Wirtz, K. W., Eckhardt, B., 1996. Effective variables in ecosystem models with an application to phytoplankton succession. Ecol. Mod. 92, 33–53.

Yuan, Y., Wei, H., Zhao, L., Jiang, W., 2008. Observations of sediment resuspension and settling off the mouth of Jiaozhou Bay, Yellow Sea. Cont. Shelf Res. 28 (19), 2630–2643.
